# Inflammatory bowel disease and COVID-19 outcomes: a meta-analysis

**DOI:** 10.1038/s41598-022-25429-2

**Published:** 2022-12-09

**Authors:** Maheeba Abdulla, Nafeesa Mohammed, Jehad AlQamish, Mahmoud Mosli

**Affiliations:** 1 Internal Medicine Department, Ibn AlNafees Hospital, Arabian Gulf University, Manama, Bahrain; 2 Salmaniya Medical Complex, Manama, Bahrain; 3 Department of Medicine, King Abdulaziz University Hospital, Jeddah, Saudi Arabia

**Keywords:** Gastroenterology, Medical research

## Abstract

There is conflicting evidence concerning the effect of inflammatory bowel disease (IBD) on COVID-19 incidence and outcome. Hence, we aimed to evaluate the published evidence through a systematic review process and perform a meta-analysis to assess the association between IBD and COVID-19. A compressive literature search was performed in PubMed/Medline, Scopus, Embase, and Cochrane Library from inception to July 2021. A snowball search in Google, Google Scholar, Research Gate, and MedRxiv; and bibliographic research were also performed to identify any other relevant articles. Quantitative observational studies such as cohort, cross-sectional, and case–control studies that assessed the incidence, risk, and outcomes of COVID-19 among the adult IBD patients published in the English language, were considered for this review. The incidence and risk of COVID-19, COVID-19 hospitalization, the severity of COVID-19, and mortality were considered as the outcomes of interest. The Joanna Briggs Institute critical appraisal checklist was used for quality assessment. A subgroup and sensitivity analysis were performed to explore the heterogeneity and robustness of the results, respectively. A total of 86 studies out of 2828 non-duplicate records were considered for this meta-analysis. The studies were single or multicentric internationally from settings such as IBD centres, medical colleges, hospitals, or from the general public. Most of the studies were observed to be of good quality with an acceptable risk of bias. The pooled prevalence of COVID-19, COVID-19 hospitalization, severe COVID-19, and mortality in the IBD population were 6.10%, 10.63%, 40.43%, and 1.94%, respectively. IBD was not significantly (p > 0.05) associated with the risk of COVID-19, COVID-19 hospitalization, severe COVID-19, and mortality. In contrast, ulcerative colitis was significantly associated with a higher risk of COVID-19 (OR 1.37; p = 0.01), COVID-19 hospitalization (OR 1.28; p < 0.00001), and severe COVID-19 (OR 2.45; p < 0.0007). Crohn’s disease was significantly associated with a lesser risk of severe COVID-19 (OR 0.48; p = 0.02). Type of IBD was a potential factor that might have contributed to the higher level of heterogeneity. There was a significant association between ulcerative colitis and increased risk of COVID-19, COVID-19 hospitalization, and severe COVID-19 infection. This association was not observed in patients with Crohns' disease or in those diagnosed non-specifically as IBD.

## Introduction

The world is still dealing with a pandemic that was first reported in Wuhan province in December 2019 and the etiological agent was recognized as severe acute respiratory syndrome coronavirus 2 (SARS CoV2)^1^. During this period of one and half years (till 31st August 2021), a total of 217,925,862 cases were identified with a total of 4,524,091 death cases reported across the world^2^. Many factors including advanced age, obesity, and comorbidities such as diabetes mellitus, hypertension, epilepsy, sarcopenia and schizophrenia were associated with severe COVID-19^3–7^. Moreover, damages to the internal organs such as the heart, liver, and kidneys were identified as major factors linked with severe COVID-19. Other factors such as time to hospital admission, tuberculosis, inflammation disorders, and coagulation dysfunctions also contributed to the higher fatality and mortality in COVID-19 patients^3^. Factors like current malignant status, dyspnoea, neutrophil/lymphocyte ratio, elevated C-reactive protein, lactate dehydrogenase, creatinine levels, oxygen saturation at admission, and use of azithromycin have been associated with mortality in geriatric COVID-19 patients^8^.

Inflammatory Bowel Diseases (IBDs) are a group of chronic conditions which affect the small and large intestines. Sustained inflammation in the gut may contribute to permanent damage of the intestine, compromise the quality of life and increase healthcare costs^9^. In 2017 a total of 6·8 million (6.4–7.3) people were reportedly living with IBD worldwide. Between 1990 and 2017, the age-standardized prevalence rate of IBD hiked from 79.5 (75.9–83.5) to 84.3 (79.2–89.9) persons per 100,000 population, while the death rate decreased from 0.61 (0.55–0.69) to 0.51 (0.42–0.54) per 100,000 population^10^.

The incidence and risk of COVID-19 in patients with IBD is still inconclusive. Several studies indicate a higher risk of COVID-19 and mortality in patients with IBD along with other factors such as advanced age and comorbidities^11,12^. In contrast to this, studies conducted by Maconi et al.^13^ and Ardizzone et al.^14^ reported a lower risk of COVID-19 in IBD patients. Interestingly, several other studies observed no cases of COVID-19 in the IBD cohorts they investigated^15–18^. In presence of all these conflicts, we aimed to identify all the currently available literature and assess the risk and outcomes of COVID-19 in patients with IBD through a comprehensive systematic literature review process and meta-analysis.

## Methodology

We followed a PECOS framework (Population, Exposure, Control, Outcome, and Study Design) for the inclusion of relevant studies and adapted the Preferred Reporting Items for Systematic Reviews and Meta-Analyses (PRISMA) Guidelines^19^ to report this systematic review. Two independent reviewers were involved in the study selection, data extraction and methodological quality assessment and any disagreements were resolved through discussion or consultation with another reviewer.

### Criteria for considering the studies for this review

#### Participants

We only considered patients who were diagnosed with IBD (CD, UC or IBD-unclassified) or microscopic colitis (MC) as per the author’s discretion in adult patients. Studies involving patients aged less than 18 or a population that included any disease other than IBD were excluded.

#### Exposure

The exposure or the etiology of interest were diagnosed with IBD such as CD, UC or IBD-unclassified and MC as per the author’s discretion.

#### Control

The comparator group considered were those who did not have IBD in the case of cohort studies and non-COVID patients in the case of case–control studies.

#### Outcomes

The outcomes considered were incidence and risk of COVID-19, COVID-19 hospitalization, severity of COVID-19, and mortality. Any studies which did not provide outcomes that were specific to IBD patients were excluded. The outcomes were considered as per author’s discretion or based on the report from authors.

#### Study designs

The quantitative observational studies such as cohort, cross-sectional (Descriptive and analytical), and case–control studies that assessed the incidence, risk, and/or outcomes of COVID-19 among adult IBD patients were considered for this review. The descriptive cross-sectional studies which presents only the prevalence were termed as prevalence studies and analytical cross-sectional studies marked as cross-sectional studies. Only the studies with full text available in the English language were considered. Reviews, descriptive studies, clinical trials, commentary, guidelines, and qualitative analyses were excluded.

### Search methods for identification of studies

A comprehensive literature search was performed in PubMed/Medline (https://pubmed.ncbi.nlm.nih.gov/), Scopus (https://www.scopus.com/), Embase (https://www.embase.com/#search) and Cochrane Library (https://www.cochranelibrary.com/advanced-search) using all the possible keywords and entry terms in July 2021. We also did a snowball search in Google, Google Scholar Research Gate, and MedRxiv (https://www.medrxiv.org/) to identify any relevant articles. The reference lists of potential articles were also screened to identify additional potentially relevant citations. A detailed search strategy in various databases is provided as Supplementary File S1.

### Study selection

All articles identified from databases following the literature search were retrieved to an Excel sheet and screened against the pre-defined criteria. The studies were screened by first reading the title and abstracts followed by reviewing the full text. Only studies that were not excluded at this stage were considered for final inclusion in the review.

### Data extraction

The data were abstracted to a comprehensive data extraction form by two independent reviewers. The author’s first name and year of publication were used to identify the studies. The data regarding the publication, study settings, participants, and outcomes were captured from the studies. The number of events and sample size were collected from the studies or calculated from the available data.

### Risk of bias and quality assessment

The Joanna Briggs Institute critical appraisal checklist was used to assess the methodological quality and risk of bias of included prevalence studies, cross-sectional studies, case–control studies and cohort studies^20^. The Joanna Briggs appraisal for prevalence studies addresses the appropriateness of sample frame, study participants, sample size, measure of condition, study setting, data analysis, and response rate. The checklist for cross-sectional studies assessed study aspects such as inclusion criteria, study subjects and setting, measure of exposure, measurement of the condition, confounding factors, outcomes measurement, and the statistical analysis used. The checklist for the case–control studies assessed study aspects such as comparability and matching of population, participant criteria, measurement of exposures, confounding factors, strategies to deal with confounding factors, outcome measurement, follow-up time, and statistical analysis The checklist for the cohort studies assessed study aspects such as recruitment of population, group assignment, measurement of exposures, confounding factors, strategies to deal with confounding factors, outcome measurement, follow-up time, incomplete data, and statistical analysis^20^.

### Evidence synthesis and meta-analysis

All the evidence extracted through the systematic process was summarized narratively and presented in tabular form. Review Man 5.3 was used to conduct the meta-analysis^21^. The number of events and the total number of participants was used calculate prevalence rates and results were presented in terms of percentage with the 95% confidence interval (CI). The odds ratio (OR) was captured or calculated for the risk outcomes and the results were presented in terms of OR with 95% CI. The I^2^ statistics were used to estimate the heterogeneity in the analysis. We used the random effect model in case of substantial heterogeneity (I^2^ > 50%; P < 0.10) during all analyses. To explore the sources of heterogeneity, we performed subgroup analysis based on the type of IBD, wherever possible^22^.

### Publication bias and sensitivity analysis

Visual inspection of the funnel plot generated through RevMan 5.3^21^ was used to analyse the publication bias wherever feasible, i.e., analyses with minimum of 10 studies^22–24^. Whereas, statistical tests such as Egger’s and Begg’s test using comprehensive meta-analysis (trial version) were performed for all the analyses to check the statistical significance of publication bias. A probability of less than 0.05 was considered statistically significant. The sensitivity analysis was performed to check the robustness of the findings by removing the study with the lowest weight in each analysis and results were provided^22^.

## Results

### Study selection process

**Figure 1:**
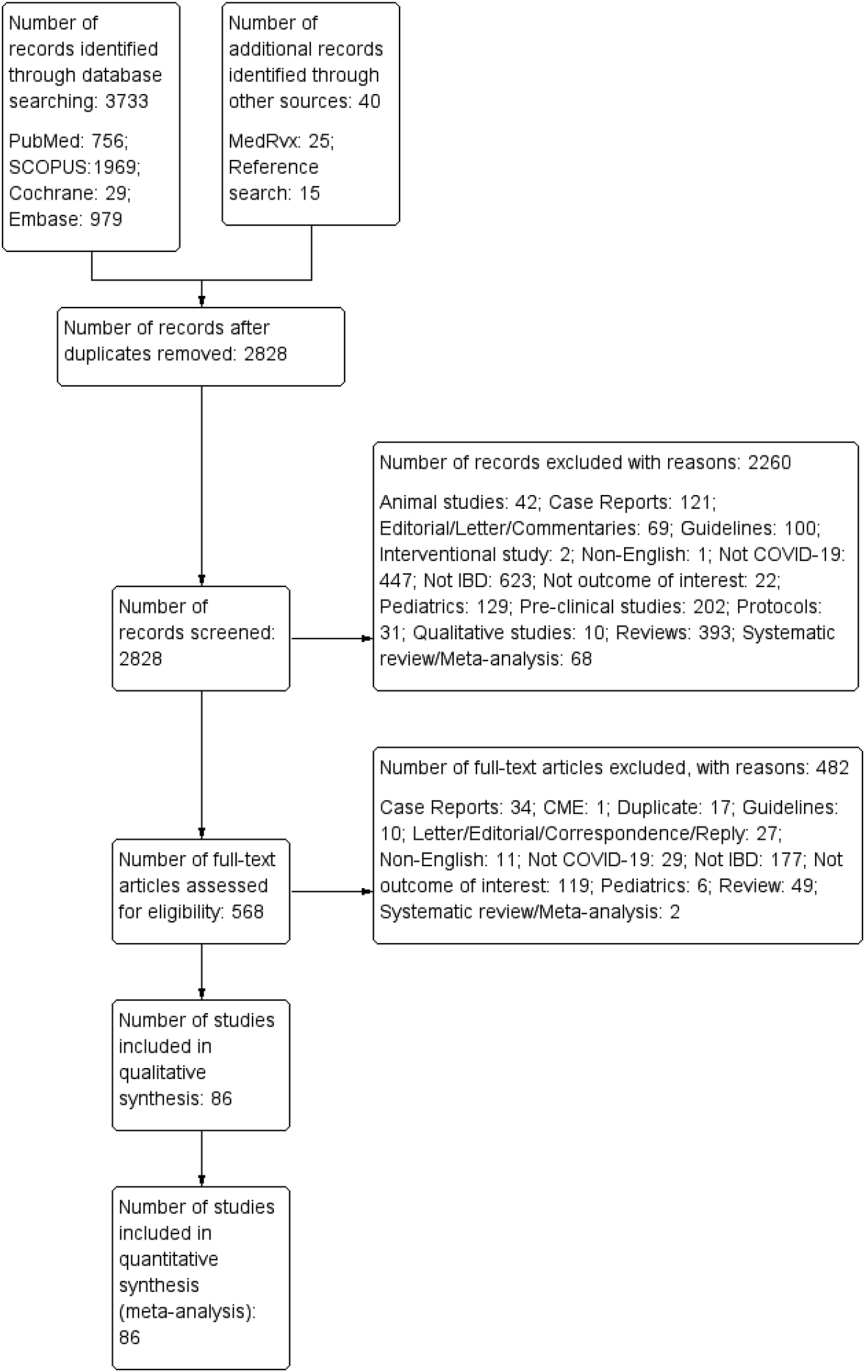
Study selection process.

A total of 3733 records were identified from the electronic databases and 40 articles from other resources. Then, a total of 2828 non-duplicate records were initially screened by their title and abstracts, in which 2260 studies were excluded for appropriate reasons. The remaining 568 full-text articles were screened for their eligibility and 86 studies^11–14,25–106^ were considered for final inclusion in this systematic review and meta-analysis. A detailed study selection is depicted in Fig. 1.

### Characteristics of included studies

Among the included studies, 50 studies (58.1%) were published in 2021 and 36 studies (41.9%) in 2020. The majority of studies emerged from the USA (30.23%; n = 26), followed by Italy (20.93%; n = 18), Spain (13.95%; n = 12), Denmark (3.49%; n = 3), France (3.49%; n = 3), the United Kingdom (3.49%; n = 3) Germany (2.32%; n = 2), Iran (2.32%; n = 2), Israel (2.32%; n = 2), and Norway (2.32%; n = 2). The remaining studies were from countries such as Chile (1.16%; n = 1), Egypt (1.16%; n = 1), China (1.16%; n = 1), France & Italy (1.16%; n = 1), India (1.16%; n = 1), Italy & Germany (1.16%; n = 1), Netherlands (1.16%; n = 1), Poland (1.16%; n = 1), Romania (1.16%; n = 1), Saudi Arabia (1.16%; n = 1), Serbia (1.16%; n = 1), and Sweden (1.16%; n = 1); information regarding the country was not available from one study (1.16%; n = 1) as it was a conference abstract. The studies included were single or multi centre; national or international; retrospective or prospective cohort studies, population-based cohort studies, case–control studies, registry analysis, and direct or web-based cross-sectional studies. A total of 34,059,455 participants were included among the studies, out of which 814,633 were IBD patients. The data were collected from IBD centres, medical colleges, hospitals or from the general public. A detailed assessment of characteristics for each included study is illustrated in Table 1.

**Table 1:** Characteristics of the included studies and patients.

Study ID, year, country	Study design; study settings and duration	Total number of participants in the study	Type of IBD	Age of IBD cohort	Male/female/other in IBD cohort	COVID-19 outcomes reported
Marafini I et al., 2020; Italy	Observational study; Tertiary referral centre; March 24 to April 30, 2020	672	CD: 397; UC: 269; IBD-U: 6	46 (16–83)^b^	311 (46)/361 (54)	Prevalence of COVID-19, COVID-19 Hospitalization, Mortality
Santervás et al., 2021; Spain	Retrospective cohort study; endoscopic database (Endobase, Olympus), medical records (Jimena IV) and the clinical reports system in hospital; March 1st to August 31st in 2019 and 2020	22	UC: 22	March–Aug 2019: 52 (27); March-Aug 2020: 63 (53)^a^	11 (50)/11 (50)	Prevalence of COVID-19, COVID-19 Hospitalization, Mortality
Attauabi et al., 2021; Denmark	Population-based cohort (The Danish COVID-IMID cohort) study; Capital Region of Denmark and Region Zealand; January 28- September 15, 2020	2,734,422 with 19,047 IBD patients	UC: 11,882; CD: 7165	NS	NS	Prevalence of COVID-19
Moum et al., 2021; Norway	Cross-sectional self-report questionnaire study; Outpatient clinic at Oslo University Hospital; June 18-September 18 2020	522 IBD patients	CD: 317; UC: 205	UC: 39.84 (13.52); CD: 41.46 (15.53)^a^	UC: 129 (62.9)/76 (37.1); CD: 172 (54.3)/145 (45.7)	Prevalence of COVID-19; COVID-19 Hospitalization
Ferrer et al., 2020; Spain	E-mail based survey; IBD Unit; March 16 to May 2, 2020	30 out of 164 IBD patients	NS	NS	NS	Prevalence of COVID-19
Lamb CA et al., 2021; UK	National multicentre observational cohort study; 1 March 2020 and 31 August 2020	211 IBD patients	CD: 109; UC: 86; IBD-U: 16	Non-Severe COVID-19: 55.0 (37.0–71.0); Severe COVID-19: 73.0 (59.8–81.0)^b^	Non-severe COVID-19: 80 (51.6)/74 (47.7); Severe COVID-19: 36 (64.3)/20 (35.7)	Risk of severe COVID-19
Richter et al., 2021; Israel	Anonymous questionnaire survey; November 2020 until January 2021	2152 IBD patients	CD: 1337; UC: 815	39 (28–51)^b^	849 (39.5)/1303 (60.5)	Prevalence of COVID-19, Risk of COVID-19, COVID-19 Hospitalization
Belleudi V et al., 2021; Italy	Retrospective Cohort Study; regional administrative healthcare databases; 31 December 2019–15 December 2020	22,525 IBD patients and 4,702,567	NS	< 50: 41.3%; 50–60: 22.1%; 60–70: 17.4%; 70–80: 12.4%; > = 80: 6.9%	11,713(52)/10,812 (48)	Prevalence of COVID-19, Risk of COVID-19, Risk of hospitalization; Risk of Death
Caron B et al., 2021; France	Cross sectional survey; University Hospital; January 8th to February 22nd, 2021	104 IBD patients	CD: 77; UC: 27	42 (13.2)^a^	50 (48.1)/54 (51.9)	Prevalence of COVID-19
Sperger J et al., 2021; USA	Cross sectional study; SECURE-IBD database; March–October 2020	2,709 IBD with COVID	CD: 1516 (56)/UC: 1132 (41.8)	41.2 (18.0)^a^	1339 (49.4)/1326 (48.9)/1 (0)	COVID-19 Hospitalization; Death
Ben-Tov A et al., 2021; Israel	Retrospective cohort study; Maccabi Healthcare Services central computerized database; Till June 23 2021	IBD: 12,231; Matched cohort: 36,254	CD: 5422; UC: 6339; IBD-U: 452	IBD: 47 (17); Matched cohort: 47 (17)^a^	IBD: 6124 (50.1)/6089 (49.9); Matched cohort: 18,135 (50)/18,120 50.0	Prevalence of COVID-19; Risk of COVID-19
Markovic S et al., 2021; Serbia	Observational study; University Hospital; October 2020	287 IBD patients receives biologic therapy	CD: 181; UC: 106	45 (18)^a^		Prevalence of COVID-19, severity of COVID
Botwin G et al., 2021; USA	Cross sectional survey; Prospective, nationwide registry; Till March 22, 2021	246 IBD	CD: 165; UC: 81	47.4 (15.5)^a^	89 (36.2)/139 (56.5)/1 (0.4)	Prevalence of COVID-19
Mahmud N et al., 2021; USA	Case crossover study; Established Veterans Affairs cohort; March 1 2020- 30 March 2021	428 IBD	CD: 195; UC: 233	69 (58, 74)^b^	402 (93.9)/6 (6.1)	Prevalence of COVID-19
Meyer A et al., 2021; France	French nationwide study; French national health data system; 15 February to 31 August 2020	268,185 IBD	NS	50 (37–63)^b^	126,210 (48.1)/141,975 (52.9)	COVID-19 Hospitalization, Death
Berte R et al., 2021; Italy and Germany	Prospective cohort study; Different geographical areas of Italy and in Germany; April to June 2020	354 IBD and 129 Controls	CD: 216; UC: 132; IBD-U: 6	IBD: 43 (31–57)/Control: 45 (35–60)^b^	IBD: 220 (62.5)/134 (37.5); Control: 84 (65)/45 (35)	Prevalence of COVID-19; Risk of COVID-19
Khan N (1) et al., 2021; USA	Nationwide retrospective cohort study; US Veterans’ Affairs healthcare system; January 1, to June 30, 2020	38,378 IBD and 67,433 without IBD	NS	IBD: < 50 years: 7,885 (20.55); 50–65 years: 9,945 (25.91); > 65 years: 20,548 (53.54); Non-IBD: < 50 years: 13,839 (20.52); 50–65 years: 17,359 (25.74); > 65 years: 36,235 (53.73)	IBD: 35,093 (91.44)/3,285 (8.56); Non-IBD: 61,464 (91.15)/5,969 (8.85)	Prevalence of COVID-19; Risk of COVID-19
Álvarez PF et al., 2021; Spain	Observational Online survey; Association of Crohn’s Disease and Ulcerative Colitis; April and June 2020	168 IBD	CD: 107; UC: 54; IBD-U: 7	< 20 years: 9; 21–40 years: 70; 41–60 years: 74; > 60 years: 15	81 (48.2)/87 (51.8)	Prevalence of COVID-19
Rottoli M et al., 2021; Italy	Retrospective study; Seven referral centres across five European countries; 9 March to 30 June 2020	91 IBD who need surgery	CD: 54; UC: 37	43 (17.1)^a^	59 (64.8)/32 (35.2)	Prevalence of COVID-19
Refaie et al., 2021; Spain	Prospective, single-centre and analytical observational survey; IBD unit ENEIDA database; August 2020	426 IBD patients	CD: 177; UC: 249	18–24 years: 3; 25–34 years: 43; 35–44 years: 86; 45–54 years: 111; 55–64 years: 99; 65–80 years: 84	209 (49.1)/217 (50.9)	Prevalence of COVID-19
Newsome RC et al., 2021; USA	Prospective observational study; University of Mississippi Medical Centre; April to July 2020	93 patients	NS	COVID: 62.3 (13.4); COVID recovered: 46.7 (16.1); Non-COVID: 55.0 (15.8)^a^	COVID: 28 (56)/22 (44); COVID recovered: 4 (44)/5 (56); Non-COVID: 14 (41)/20 (59)	Risk of COVID-19
Navarro-Correal et al., 2021; Spain	Retrospective study; Reference hospital in Spain; 2 March to 17 April 2020	556 IBD patients	CD: 331; UC: 225	CD: 43.18 (13.95); UC: 47.52 (13.21)^a^	CD: 43.18 (13.95)/171 (51.7); UC: 47.52 (13.21)/115 (51.1)	Prevalence of COVID-19
Gubatan J et al., 2020; USA	Retrospective analysis; Stanford Clinic; March 04, to April 14, 2020	168 IBD patients	CD: 66; UC: 86; IBD-U: 6	47.7 (16.3)^a^	80 (47.6)/88 (52.4)	Prevalence of COVID-19; Severity of COVID-19
Rodríguez-Lago I et al., 2020; Spain	Cross-sectional study; online database using REDCap; February 27 to April 8, 2020	40 COVID-19 IBD patients	CD: 13; UC: 23; IBD-U: 4	59 (48–68)^b^	24 (60)/16 (40)	COVID-19 Hospitalization; Death
Brenner EJ et al., 2020; USA	Cross sectional study; International Registry SECURE-IBD study; NS	525 Patients	CD: 312; UC: 203; IBD-U: 7; Missing: 3	42.9 (18.2)^a^	276 (52.6)/243 (46.3)/6 (1.1)	COVID-19 Hospitalization; Death; Risk of hospitalization, Risk of death
Allocca M (1) et al., 2020; France and Italy	Cohort study; Nancy and Milan Cohorts; NS	15 COVID patients in 6000 IBD patients	CD: 8; UC: 7	NS	4 (26) /11 (74)	Prevalence of COVID-19; COVID-19 Hospitalization; Death
Scaldaferri F et al., 2020; Italy	Observational prospective study; IBD Centre; 4 March to 15 April 2020	1451 IBD patients receiving biologic therapy	CD: 784; UC: 522; IBD-U: 87; Pouchitis: 87	44 (15)^a^	842 (58)/609 (42)	Prevalence of COVID-19
Taxonera C (1) et al., 2020; Spain	Single-centre, observational case-series; IBD Unit in the Madrid region; Till April 8 2020	1918 IBD patients	CD: 920; UC: 998	52 (16)^a^	997 (52)/921 (48)	Prevalence of COVID-19; Risk of COVID-19
Bezzio et al., 2020; Italy	Italian prospective observational cohort study; 24 IBD referral unit; 11 to 29 March 2020	79 COVID-19 IBD patients	CD: 32; UC: 47	45 (18–80)^b^	44 (55.7) /35 (44.3)	COVID-19 Hospitalization; Death; Risk of COVID-19; Risk of death
Quera R et al., 2020; Chile	Observational, cross-sectional and analytical study; March 1 to August 31, 2020	1432 IBD patients	COVID-IBD: CD: 14; UC: 18	32 (18–69)^b^	IBD-COVID: 14 (44)/18 (56)	Prevalence of COVID-19; COVID-19 Hospitalization
Vadan R et al., 2020; Romania	Observational study; Hospital computerized registries; 15 March to 15 August 2020	410 IBD	CD: 253; UC: 157	41 (14.2)^a^	218 (53.2)/192 (46.8)	Prevalence of COVID-19; COVID-19 Hospitalization; COVID-19 Severity
Anushiravani A et al., 2020; Iran	Management protocol study; Iranian registry of Crohn’s and colitis; NS	13,165 IBD patients	NS	NS	NS	Prevalence of COVID-19
Femury M et al., 2020; France	Cross sectional study; Amiens University Centre; March 25 to May 11, 2020	146 IBD who received biologic therapy	CD: 122; UC: 24	38 (17–81)^b^	72 (49.3)/74 (50.7)	Prevalence of COVID-19; Risk of COVID-19
Xu F et al., 2021; USA	Cross sectional study; Medicare data; April 1 to July 31, 2020	IBD: 249,406; Control: 24,254,960	CD: 96,908; UC: 152,498; Control: 24,254,960	NS	NS	Risk of Hospitalization; Risk of death
Ardizzone S et al., 2021; Italy	Observational retrospective multicentre study; February to May 2020	1816 IBD patients on biologic therapy	CD: 1177; UC: 626; IBD-U: 13	45^a^	998 (54.9)/818 (45.1)	Prevalence of COVID-19; Risk of COVID-19
Queiroz NSF et al., 2021; USA	Cross sectional study; SECURE-IBD registry analysis; March 13 to November 24, 2020	230 COVID-19-IBD patients	CD: 115; UC 114; IBD-U: 1	40.47 (16.2)^a^	92 (40)/137 (59.6)/1 (0.4)	Hospitalization; Death; Risk of hospitalization; Risk of Death
Hadi YB et al., 2021; USA	Retrospectively analysis; Multi-institutional Research Network; Till April 30, 2021	IBD: 5562; Non-IBD: 859,017	NS	IBD: 57.3 (17.5); Non-IBD: 57.9 (18.1)^a^	IBD: 2299 (41.33)/3263 (58.67) Non-IBD: 360,857 (42.01)/498,160 (57.99)	Prevalence of COVID-19; Risk of COVID-19
Crispino et al., 2021; Italy	Cross-Sectional Survey; IBD Clinic; April 5 to 15, 2021	276 IBD patients	CD: 148; CD: 128	NS	149 (54)/127 (46)	Prevalence of COVID-19; Risk of COVID-19
Agrawal M et al., 2021; USA	Cross-sectional study; SECURE-IBD database; NS	2019 COVID-19-IBD patients	CD: 1297; UC: 658; IBD-U: 52	35.7 (17.87)^a^	968 (47.9)/1051 (52.1)	COVID-19 Hospitalization; Death
Askar SR et al., 2021; Egypt	Cross-sectional study; 15 November to December 2020; Egypt	105 IBD patients	CD: 23; UC: 82	33.2 (11)^a^	49 (46.7)/56 (53.3)	Prevalence of COVID-19
Kjeldsen J et al., 2021; Denmark	Cross sectional study; Population based national register study; March 1, to October 31, 2020	52 (39.4) IBD 132 exposed; Control: 2811	NS	NS	NS	Risk of Death
Khalili et al., 2021; USA	Matched case–control study; Nationwide pathology cohort (ESPRESSO); Till February 1, 2020	MC: 10,552; Comparator: 52,624	CC: 3237; LC: 7315	CC: 65.1; LC: 64.7^a^	NS	Risk of COVID-19 hospitalization; Risk of Severe COVID-19
Taxonera C (2) et al., 2020; Spain	Single-centre, cross-sectional study; IBD referral unit; March 11 to April 8, 2020	212 IBD patients	NS	NS	NS	COVID-19 Hospitalization
Agrawal M (1) et al., 2020; USA	Cross sectional study; SECURE-IBD registry; NS	1499 COVID-19 IBD patients	CD: 821; UC: 649; IBD-U: 24	44 (17.88)^a^	747 (49.8)/752 (50.2)	COVID-19 Hospitalization
Ghoshal UC et al., 2021; India	Cross sectional study; IBD clinic; 28 May to 16 July 2020	50 IBD patients	CD: 16; UC: 34	23^b^ 23–66^c^	28 (56)/22 (44)	Prevalence of COVID-19; Severity of COVID-19
Axelrad JE et al., 2021; USA	Case series; IBD Centre; March 3 to May 10, 2020	83 IBD patients	CD: 56; UC: 27	35 (27–45)^b^	44 (53)/39 (47)	Prevalence of COVID-19; Death
Kennedy NA et al., 2021; UK	UK wide, multicentre, prospective observational cohort study; National Health Service (NHS) hospitals; 22 September to 23 December 2020	6935 IBD patients	CD: 3949; UC: 2810; IBD-U: 176	39.0 (28.7–53.2)^b^	3705 (53.4)/3221 (46.4)/9 (0.2)	Prevalence of COVID-19; COVID-19 Hospitalization
Dailey J et al., 2020; NS	Cross sectional study; NS; Till May 2020	410 IBD patients	CD: 305; UC: 105	17^a^	242 (59)/168 (41)	Prevalence of COVID-19
Agrawal M (2) et al., 2020; USA	Cross sectional study; SECURE-IBD registry; NS	3647 COVID-IBD patients on vedolizumab	CD: 2049; UC: 1527; IBD-U: 57	42.2 (16.4)^a^	1800 (49.4) /1847 (50.6)	COVID-19 Hospitalization; COVID-19 severity
Agrawal M (3) et al., 2020; USA	Cross sectional study; SECURE-IBD registry; March to September 2020	2326 COVID-IBD patients on Tofacitinib	CD: 1299; UC: 976; IBD-U: 45	41.5 (18.1)^a^	1176 (50.6)/1150 (49.4)	COVID-19 Hospitalization; COVID-19 severity; Death
Derikx et al., 2021; Netherlands	Nationwide multicentre, retrospective cohort study; Two academic and 18 non-academic hospitals; May 2019 to June 2020	100 COVID-IBD patients in 34,763 IBD cohort	CD: 36; UC: 59; IBD-U: 5	62.5 (23)^b^	46 (46)/54 (54)	Prevalence of COVID-19; COVID-19 Hospitalization; Death; Risk of COVID-19; Risk of hospitalization
Attauabi M et al., 2020; Denmark	Prospective Cohort study; Danish population; January 28, to June 2, 2020	76 COVID-IBD patients	CD: 31; UC: 45	51 (39–66)^b^	45 (59)/31 (41)	Prevalence of COVID-19; COVID-19 Hospitalization; Death; Risk of hospitalization; Risk of death
Ungaro RC et al., 2020; USA	Cross sectional study; SECURE-IBD database; 13 March to 9 June 2020	1439 COVID-IBD patients on medication	CD: 794; UC: 690	44.1 (17.6)^a^	740 (51.4)/699 (48.6)	Severity of COVID-19; COVID-19 Hospitalization; Death
Łodyga M et al., 2021; Poland	multi‑ centre, prospective, observational study; Polish IBD patients; May 1 to June 15, 2020	IBD: 432; Control group: 432	CD: 290; UC: 142	IBD: 35.7 (12.4); Control: 35.7 (12.3)^a^	IBD: 259 (60)/173 (40); Control: 259 (60)/173 (40)	Prevalence of COVID-19; Risk of COVID-19
Rizzello F et al., 2021; Italy	Observational study; Single tertiary IBD centre; March 10 to June 10 2020	1158 IBD patients	CD: 695; UC: 463	44.48 (14.56)^a^; 18–81^c^	644 (55.6)/514 (44.4)	Prevalence of COVID-19; Hospitalization; Death
Maconi G et al., 2021; Italy	Observational study; Eight major gastrointestinal centres in Lombardy, Italy; April 4, to April 12, 2020	IBD: 941; Controls: 869	NS	IBD: 50 (39–60); Control: 54 (44–63)^b^	IBD: 485 (51.5)/456 (48.5); Control: 394 (45.3)/475 (54.7)	Risk of COVID-19; Risk of hospitalization; Risk of death
Ludvigsson JF et al., 2021; Sweden	Population‐based cohort study; Nationwide registers in Sweden; 1 February to 31 July 2020	IBD: 67,292; Control: 297,910	CD: 21,599; UC: 43,622; IBD‐U: 2071	IBD: 39.1 (16.8)^a^ 37.5 (25.7–51.6)^b^; Control: 38.0 (16.2)^a^ 36.4 (25.1–49.9)^b^	IBD: 34,321 (51)/32,971 (49); Control: 150,103 (50.4)/147,807 (49.6)	Prevalence of COVID-19; Hospitalization; Death; severe COVID-19; Risk of COVID-19; Risk of hospitalization; Risk of death; Risk of Severe COVID-19
Schlabitz F et al., 2021; Germany	Anonymous multicentre web-based survey; German population; 28 April to 31 July 2020	1199 IBD patients	CD: 701; UC: 464; IBD-U: 16; MC: 7	41.3 (12.9)^a^	275 (22.9)/924 (77.1)	Prevalence of COVID-19
Burke KE et al., 2020; USA	Cohort study; Two tertiary referral hospitals in Boston; January 2019 to April 2020	5302 IBD patients	CD: 3075; UC: 2227	46.5^a^; 18–99^c^	2650 (49)/2652 (51)	Prevalence of COVID-19; Hospitalization; Death; severe COVID-19; Risk of COVID-19; Risk of Severe COVID-19
Iborra I et al., 2021; Spain	Cross-sectional observational study; Local ENEIDA registry; March 1 and April 30, 2020	234 IBD patients on biologic therapy	CD: 178; UC: 52; IBD-U: 4	NS	124 (52.9)/110 (47.1)	Prevalence of COVID-19; Severity of COVID-19
El Hajra et al., 2021; Spain	Observational study; Spanish IBD referral hospital; March 15 to May 15, 2020	510 IBD patients	CD: 303; UC: 199; IBD-U: 8	50 (40–60)^b^	252 (49.4)/258 (50.6)	Prevalence of COVID-19; Hospitalization; Severity of COVID-19
Guerra I et al., 2021; Spain	Cross-sectional, observational study; IBD unit; April 24 to May 27, 2020	805 IBD patients; 82 suspected/confirmed COVID-IBD	CD: 42; UC: 35; IBD-U: 5	46 (14)^a^	38 (46.3)/44 (53.7)	Prevalence of COVID-19; Severity of COVID-19; Death
Orlando V et al., 2021; Italy	Retrospective case–control study; Administrative health-related database; Till June 10, 2020	Cases:3,497; Controls: 17,358	NS	Cases: 30–64 years: 2,375; ≥ 65 years: 1,122; Control: 30–64 years: 11,829; ≥ 65 years: 5,538	Cases: 1,945 (55.6)/1552 (44.4); Control: 9,640 (55.5)/7718 (44.5)	Risk of COVID-19; Risk of severe COVID-19
Scucchi L et al., 2021; Italy	Prospective cohort study; IBD referral centre; May 27 to July 21, 2020	218 IBD patients	CD: 128; UC: 90	44 (19–77)^b^	118 (54.2)/100 (45.8)	Prevalence of COVID-19
Carparelli et al., 2021; Italy	Observational cohort study; Single, tertiary, IBD centre; Till January 2021	600 IBD patients; 25 IBD-COVID patients	CD: 14; UC: 11	46.5 (14.3)^a^	16 (64)/9 (36)	Prevalence of COVID-19; COVID-19 Hospitalization
Calafat M et al., 2021; Spain	Observational, retrospective cohort study; Two referral IBD centres; March 2020 to March 2021	418 elderly IBD patients	CD: 117; UC: 290; IBD-U: 11	73 (69–78)^b^	218 (52.2)/200 (47.8)	Prevalence of COVID-19; Death
Parekh R et al., 2021; USA	Cross sectional study; SECURE-IBD registry; NS	2035 COVID-IBD patients	CD: 1139; UC: 854; IBD-U: 42	42.7 (17.9)^a^	1030 (51); 1005 (49)	Severity of COVID-19
Dalal RS et al., 2021; USA	Cross sectional study; Direct survey and social media from IBD centres; December 22, 2020 to January 26, 2021	906 IBD patients	CD: 465; UC: 416; IBD-U: 22	Local: 45 (35–62); Social media: 40 (32–48)^b^	147 (16.2)/759 (83.8)	Prevalence of COVID-19
Khan N (2) et al., 2021; USA	Retrospective cohort study; Veterans Health Administration Cohort; December 18 2020 to April 20, 2021	14,697 (7321 Vaccinated and 7376 non-vaccinated) IBD patients	CD: 5616; UC: 9081	Unvaccinated: 64 (47, 73); Vaccinated: 71 (60, 75)^b^	13,543 (92.1)/1154 (7.9)	Prevalence of COVID-19; Severity of COVID-19; Death
Khan N (3) et al., 2021; USA	Retrospective cohort study; Veterans Health Administration Cohort; 20 January 2020 to 10 December 2020	30,911 IBD patients	CD: 12,736; UC: 18,175	65 (50–73)^b^	28,098 (90.9)/2813 (9.1)	Prevalence of COVID-19
Opheim R et al., 2021; Norway	Cross sectional Questionnaire survey; Outpatient university Hospital; June 18 to September 18 2020,	506 IBD patients	NS	40.78 (14.71)^a^	289 (57)/217 (43)	Prevalence of COVID-19
Norsa L et al., 2020; Italy	Observational study; Italian IBD centre; March 4 to July 10 2020	90 IBD patients on biologic therapy	CD: 55; UC: 35	NS	48 (53.3)/42 (46.7)	Prevalence of COVID-19; Risk of COVID-19
Harris RJ et al., 2020; UK	Online single point in time survey; High and moderate-risk IBD population; NS	685 IBD patients	CD: 443; UC: 211; IBD-U: 31	16–24 years: 52; 25–34 years: 104; 35–44 years: 154; 45–54 years: 139; 55–64 years: 118; 65–74 years: 76; 75 + years: 42	288 (42)/394 (57.5)/2 (0.3)	Prevalence of COVID-19
Allocca M (2) et al., 2020; Italy	Prospective, observational, international, multicentre cohort study; 12 Centres from 8 countries; 21 February to 30 June, 2020	23,879 IBD patients; 97 COVID-IBD patients	CD: 53; UC: 43; IBD-U: 1	42 (28.7–54.2)^b^	50 (52)/47 (48)	Prevalence of COVID-19; Hospitalization; Severity of COVID; Death; Risk of Severe COVID; Risk of hospitalization
Fantini MC et al., 2020; Italy	Multicentre national Questionnaire survey; Italian IBD referral centres; March 9 to April 14, 2020	4304 IBD patients	NS	NS	NS	Prevalence of COVID-19; Hospitalization; Severity of COVID; Death
Viganò C et al., 2020; Italy	Retrospective cohort study; Single IBD Centre; March to April 2020	704 IBD patients	CD: 295; UC: 409	53 (61–45)^b^	403 (57.2)/301 (42.8)	Prevalence of COVID-19; Risk of COVID-19
Allocca M (3) et al., 2020; Italy	Prospective cohort study; Single IBD centre; NS	41 IMID patients with 21 IBD patients	CD: 9; UC: 12	48 (31–57.5)^b^	17 (41)/24 (59)	Risk of hospitalization
Lukin DJ et al., 2020; USA	Matched cohort design; Non-academic hospital, SMART IBD longitudinal cohort; February 1 and April 30, 2020	119 IBD patients; 29 IBD-COVID patients	CD: 69; UC: 46; IBD-U: 4	< 40 years: 51; > 40 years: 68	53 (44.5)/66 (55.5)	Prevalence of COVID-19; Risk of COVID-19
Singh S et al., 2020; USA	Population-based retrospective cohort study; Federated health research network data; January 20, to May 26, 2020	196,403 IBD patients; 232 IBD-COVID patients	CD: 101; UC: 93; IBD-U: 38	51.2 (18.1)^a^	85 (36.6)/147 (63.4)	Prevalence of COVID-19; Risk of severe COVID-19; Risk of hospitalization
Bezzio C et al., 2020; Italy	Observational cohort study; IBD hospital; March 10 to May 3, 2020	243 IBD patients	NS	Biologic therapy: 45.9 (14.5); Not biologic therapy: 49 (16.1)^a^	142 (58.4)/101 (41.6)	Prevalence of COVID-19; Hospitalization; Severe COVID-19
Hong SJ et al., 2020; USA	Prospective Cohort Study; Academic medical centre; March 3 to May 10, 2020	83 IBD-COVID; 8277 non-IBD COVID	CD: 56; UC: 27	34 (24–47.5)^b^	33 (40)/50 (60)	Hospitalization; Severe COVID-19; Death; Risk of Hospitalization; Risk of Severe COVID-19; Risk of Death
Lewine E et al., 2020; USA	Retrospective chart review; IBD centres; March 1, to June 1, 2020	37 IBD patients	CD: 19; UC: 18	41 (15.9)^a^	19 (51.4)/18 (48.6)	Prevalence of COVID-19
Mosli M et al., 2020; Saudi Arabia	Cross-sectional survey; Social Media; 30 March to 5 April, 2020	1156 IBD patients	CD: 765; UC: 299; IBD-U: 92	< 16 years: 23; 17–40 years: 977; > 40 years: 155	607 (52.5)/549 (47.5)	Prevalence of COVID-19
Hormati A et al., 2020; Iran	Retrospective study; Single Centre; NS	150 IBD patients	NS	48.4 (11)^a^	NS	Prevalence of COVID-19
Grunert PC et al., 2020; Germany	Cross-sectional survey; IBD outpatient clinic; April 2 and 17, 2020	415 IBD patients and 116 controls	CD: 215; UC: 192; IBD-U 5; Not specified: 3	45^a^ 18–82^c^	188 (45.3)/227 (54.7)	Risk of COVID-19
An P et al., 2020; China	Cross-sectional survey; University Hospital; Dec 8, 2019 to March 30, 2020	318 IBD patients	CD: 114; UC: 204	39·2 (15–79)^b^	NS	Prevalence of COVID-19

*CD* Crohn’s disease, *CC* collagenous colitis, *IBD* inflammatory bowel disease, *IBD-U* IBD-Unclassified, *IMID* immune-mediated inflammatory disease, *LC* lymphocytic colitis, *MC* microscopic colitis, *NS* not specified, *UC* ulcerative colitis, *UK* United Kingdome, *USA* United States of America.

^a^Mean with standard deviation.

^b^Median with IQR.

^c^Range.

### Risk of bias in the included studies

The quality assessment or risk of bias of included studies for the prevalence and outcomes of COVID-19 patients with IBD is presented in Supplementary File S2. We did not provide a score to the studies as Joanna Briggs's guidance discourages the use of a score cut-off for quality assessment^107^. Most of the studies were observed as being of good quality with an acceptable risk of bias. Among the prevalence studies, some studies failed to report the method used for the identification of the condition and its reliability. In the case of cross-sectional studies, the risk of bias was attributed to factors such as the identification and dealing with confounding factors in the study. The methodological quality of cohort studies was observed to be good and free of bias.

### COVID-19 in patients with IBD

#### Prevalence of COVID-19

A pooled estimate of 63 studies^11,12,14,25,29,31,32,36,37,39–43,49–54,58,59,61,66–69,72,75,77–82,85,86,88–96,98,99,102–104,106^ indicated an overall prevalence rate of 6.10% (95% CI 3.15–9.04%) of COVID-19 in patients with any IBD. Heterogeneity was very high (I^2^: 100%); hence a random effect model was used.

**Figure 2:**
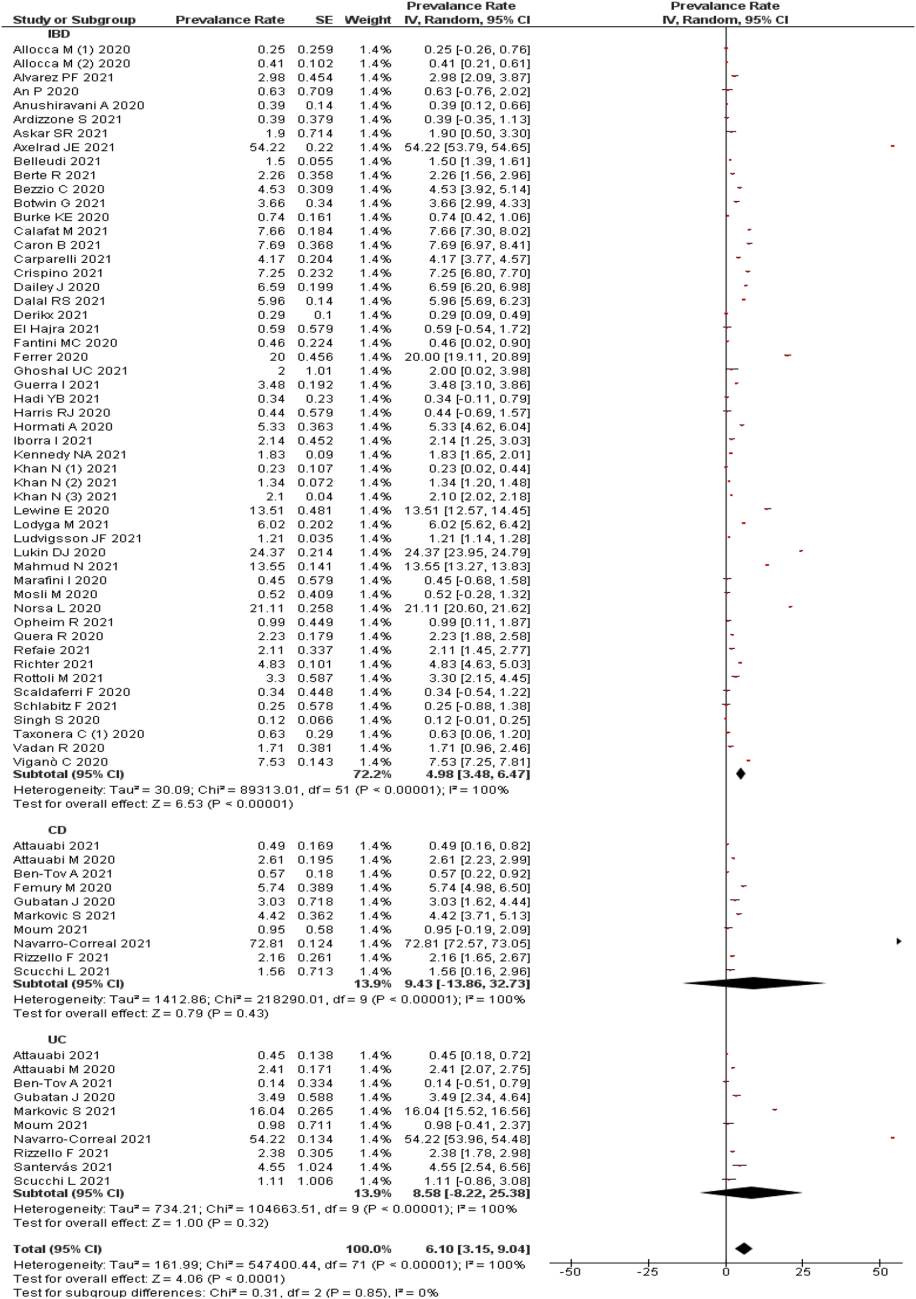
Prevalence of COVID-19 in patients with IBD.

Subgroup analysis indicated a prevalence of 9.43% (95% CI − 13.86 to 32.73%; n = 10 studies) of COVID-19 among the patients with CD^26–28,34,35,45,46,73,76,84^ and 8.58% (95% CI − 8.22 to 25.38; n = 10 studies) among those patients with UC^27,28,34,35,45,46,55,73,76,84^. This analysis indicated that, type of IBD was not a contributing factor to the heterogeneity as there was no change in the level of heterogeneity even after a subgroup analysis (Fig. 2).

Visual inspection of funnel plot observed an obvious asymmetry (Supplementary Fig. S3A) indicating the chances of publication bias which was confirmed by Begg’s test (p = 0.014), but not with Egger’s test (p = 0.087). A sensitivity analysis by removing a study by Singh et al.^99^ indicated no much difference from the overall pooled estimate (6.07%; 95% CI 3.09–9.06%; 62 studies). The result is provided in Supplementary Fig. S4A.

#### Risk of COVID-19

A meta-analysis of 22 studies^11–14,31,34,39,44,51,55,58,59,72,75,77,79,83,89,91,92,96,98,105^ indicated a non-significant association between the IBD and COVID-19 (OR 1.15; 95% CI 0.97–1.37; p = 0.11) compared to non-IBD patients. A significant heterogeneity (I^2^: 90%) was observed, hence a random effect model was applied.

**Figure 3:**
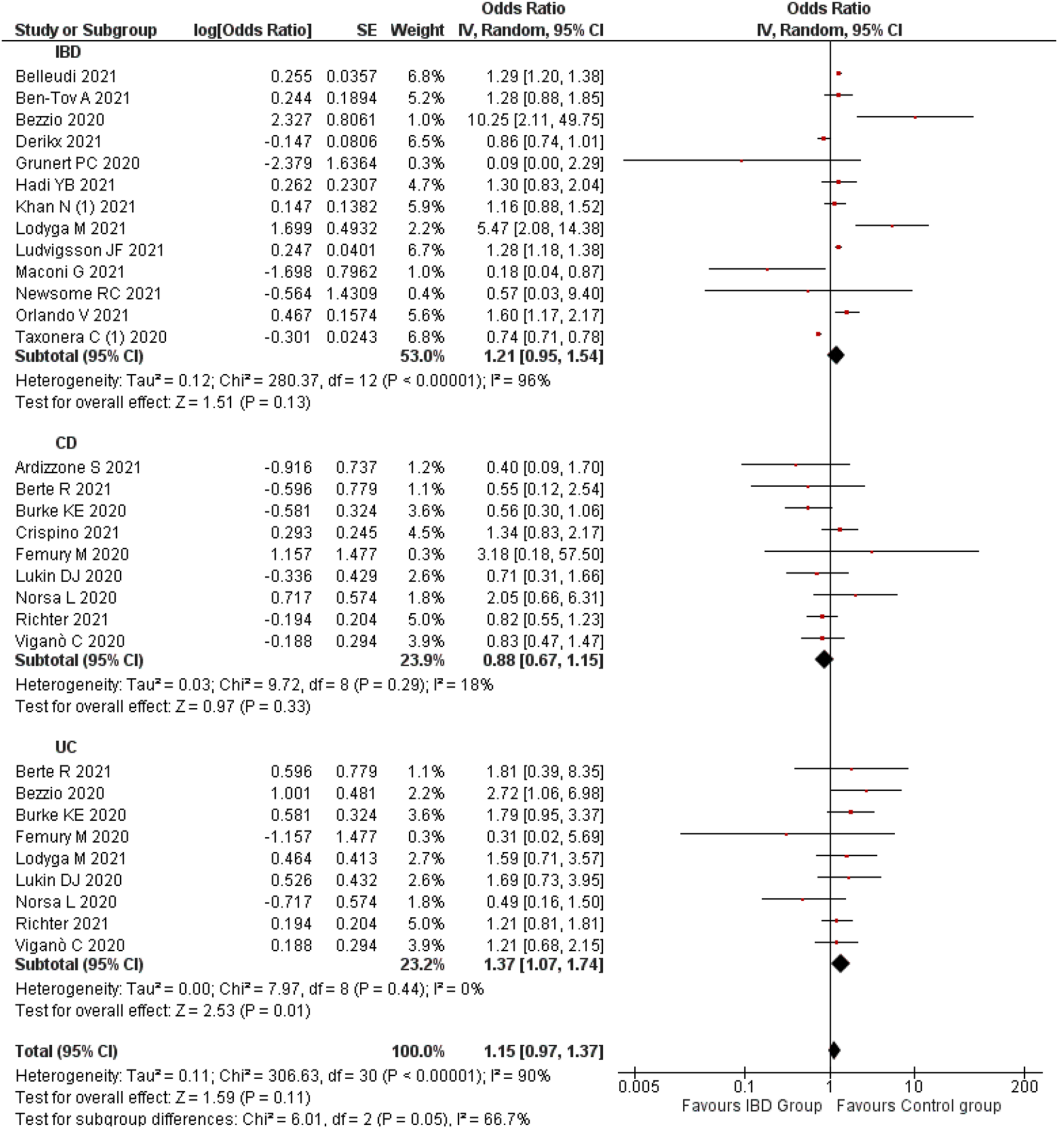
Risk of COVID-19 among the IBD patients.

A Subgroup analysis by type of IBD revealed similar non-significant association with CD patients (OR 0.88; 95% CI 0.67–1.15; p = 0.33; n = 9 studies)^14,31,39,55,59,79,92,96,98^. Whereas, UC was significantly associated with a higher risk of COVID-19 (OR 1.37; 95% CI 1.07–1.74; p = 0.01; n = 9 studies)^11,39,55,75,79,91,92,96,98^ compared to the non-UC patients. However, the heterogeneity was not observed or non-significant when we performed a subgroup analysis based on the type of IBD such as CD (I^2^: 18%) and UC (I^2^: 0%). This indicates that the type of IBD might have contributed to the variation observed among the study findings (Fig. 3).

Visual inspection of funnel plot does not show an obvious asymmetry which is suggestive no publication bias (Supplementary Fig. S3B) which was further confirmed by Egger’s (p = 0.999) and Begg’s test (p = 0.649). A sensitivity analysis by removing single study by Grunert et al.^105^ estimated no changes in the actual results (OR 1.16; 95% CI 0.97–1.38; 21 studies). The results are provided in Supplementary File S4B.

### COVID-19 hospitalization among the patients with IBD

#### Prevalence of COVID-19 hospitalization

A total of 32 studies^11,25,27,31,33,34,37,38,47–49,51–53,57,60,65,67,68,70–72,74,76,77,79,81,85,94,95,100,101^ reported COVID-19-related hospitalizations among IBD patients and a pooled estimate identified that 10.63% (95% CI 6.67–14.60%) of IBD patients were admitted to the hospital. There was high heterogeneity (I^2^: 100%) observed among the studies and random effect model was applied.

**Figure 4:**
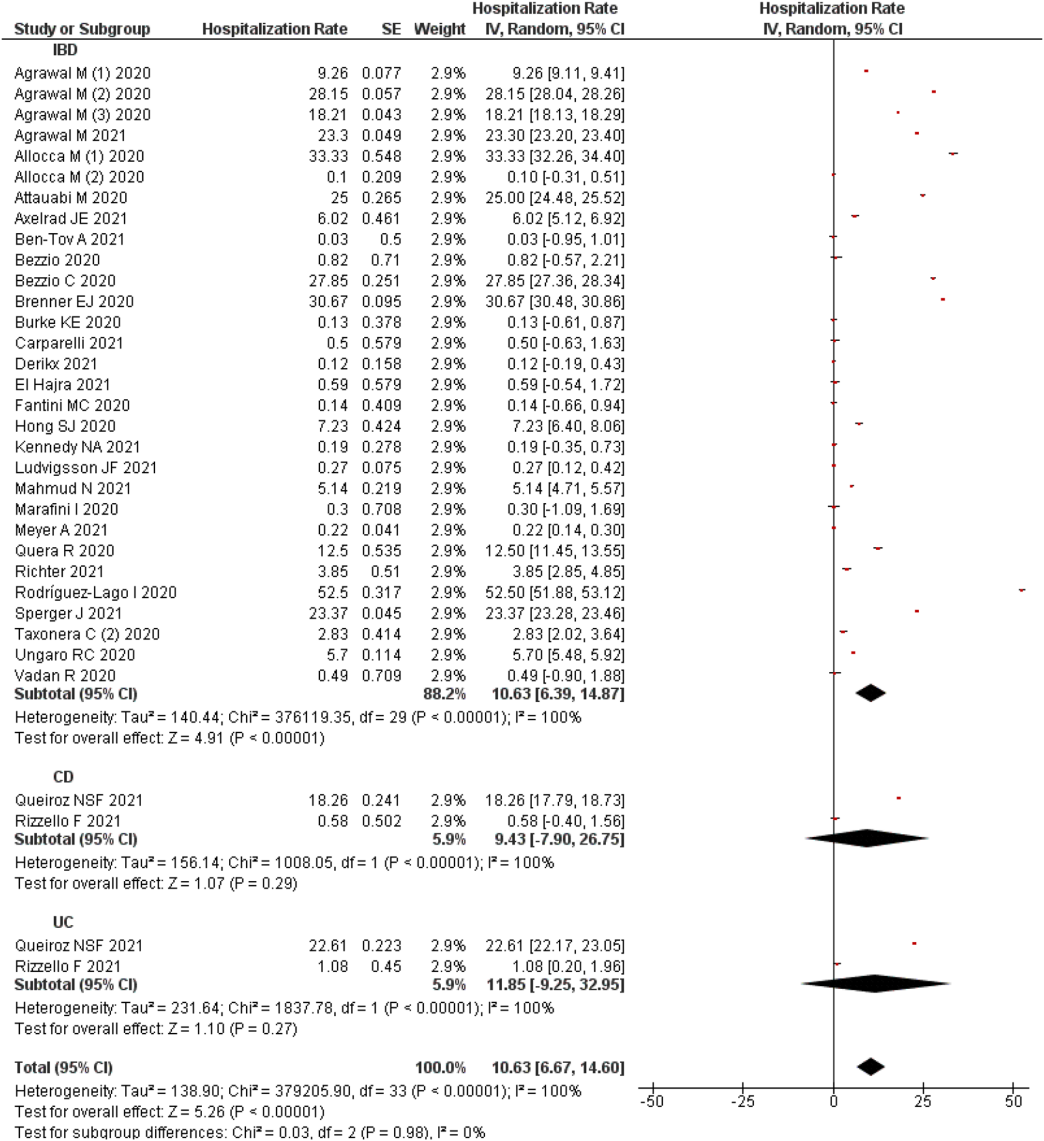
COVID-19 hospitalization among the patients with IBD.

Subgroup analysis observed that, only two studies^57,76^ recorded the COVID-19-related hospitalization rate in patients with CD and UC separately, which was 9.43% (95% CI − 7.90 to 26.75%) and 11.85% (95% CI − 9.25, 32.95%), respectively. No change was in heterogeneity was observed after subgroup analysis based on the type of IBD (Fig. 4).

Visual inspection of funnel plot observed an obvious asymmetry suggestive of publication bias (Supplementary File S3C) which was not confirmed through Egger’s (p = 0.907) and Begg’s (p = 0.252) test. A sensitivity analysis by removing single study estimated no much changes in actual results (10.94; 95% CI 6.92, 14.96; 31 studies). The results are provided in Supplementary File S4C.

#### Risk of COVID-19 hospitalization

A pooled estimate of 13 studies^12,13,27,48–50,57,63,72,77,94,99,101^ indicated that IBD (OR 1.08; 95% CI 0.87–1.33; p = 0.50) was not significantly associated with risk of COVID-19 associated hospitalization. A random-effect model was used for the analysis as there was significant heterogeneity (I^2^: 87%).

**Figure 5:**
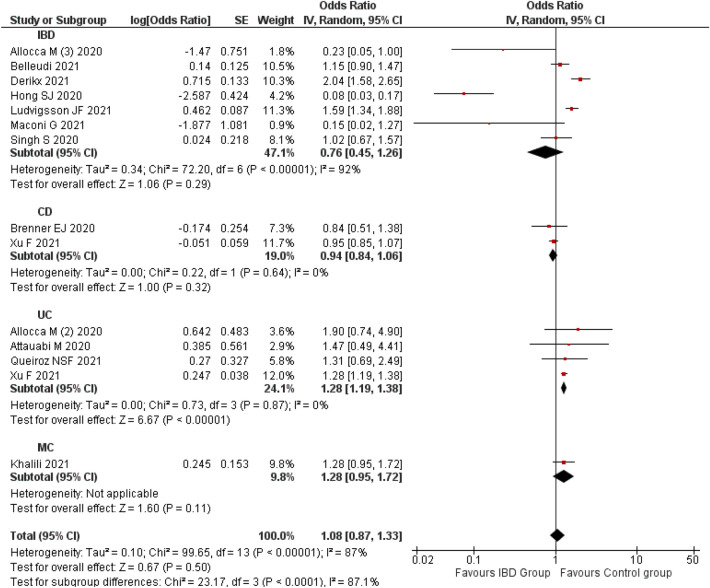
Risk of COVID-19 hospitalization among the patients with IBD.

The subgroup analysis recorded a significantly higher risk of COVID-19-related hospitalization in UC patients (OR 1.28; 95% CI 1.19–1.38; p < 0.00001; n = 4 studies) compared to non-UC patients^27,50,57,94^. However, CD (OR 0.94; 95% CI 0.84–1.06; p = 0.32; n = 2 studies)^48,50^ and MC (OR 1.28; 95% CI 0.95–1.72; p = 0.11; 1 study)^60^ was not significantly associated with risk of COVID-19 associated hospitalization. There was a non-significant heterogeneity after the subgroup analysis based on the type of IBD such as CD and UC (I^2^: 0%). This indicates that type of IBD might be a significant factor that contributed to the variation observed among the study findings (Fig. 5).

The visual inspection of funnel plot observed an obvious asymmetry (Supplementary File S3D) suggestive of publication bias which was not confirmed by Egger’s (p = 0.325) and Begg’s (p = 0.228). A sensitivity analysis by removing Maconi et al.^13^ observed no changes in the actual findings (OR 1.10; 95% CI 0.89–1.36; 12 studies). The result is provided in Supplementary File S4D.

### Severity of COVID-19 in patients with IBD

**Figure 6:**
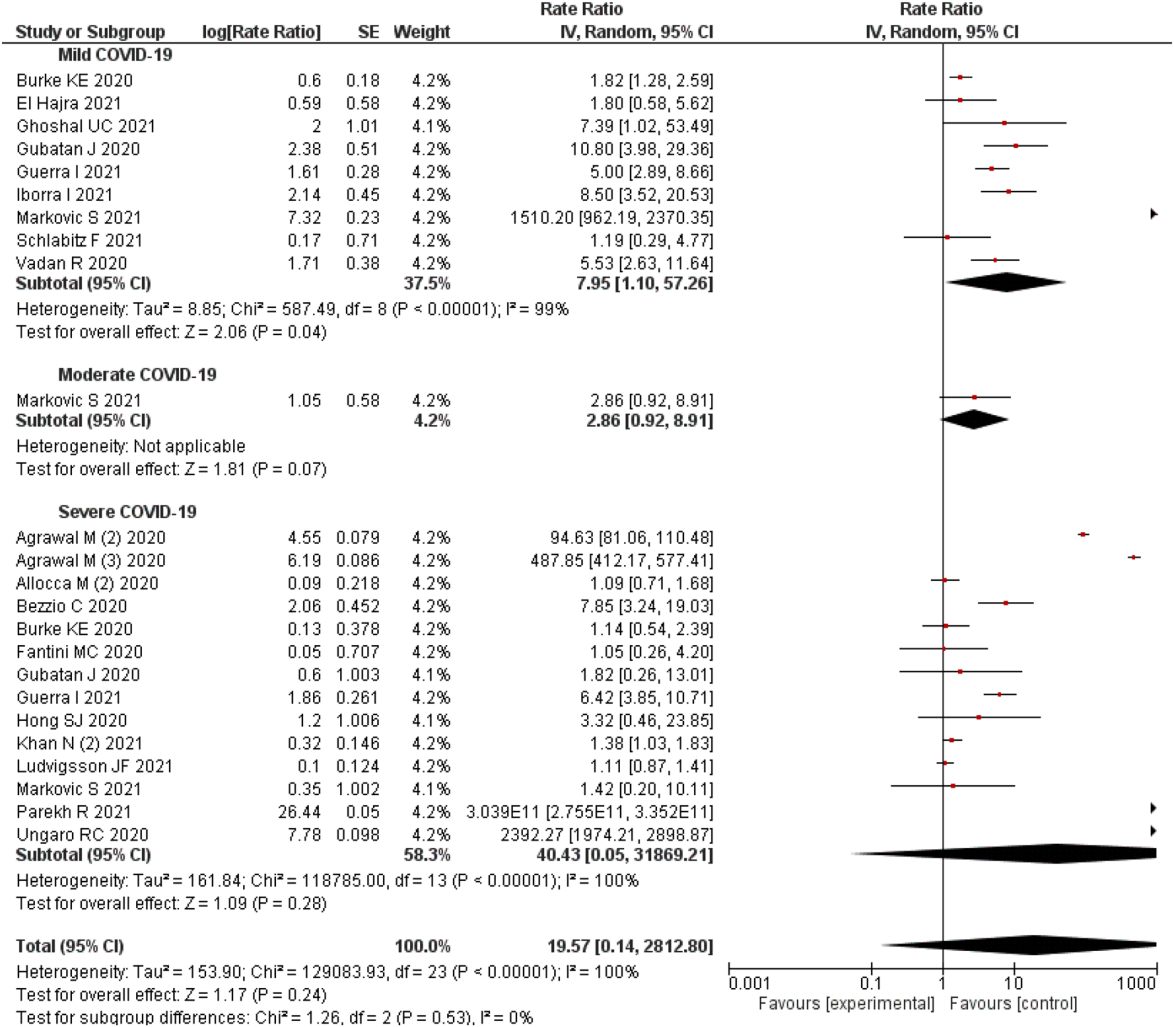
Severity of COVID-19 among IBD patients.

The severity of COVID-19 among IBD patients was reported in 19 studies^11,35,46,49,53,65,66,70,74,77–82,87,89,95,101^. The meta-analysis of these studies recorded that 7.95% (95% CI 1.10–57.26%; n = 9 studies) had mild disease^35,46,53,66,78–82^, 2.86% (95% CI 0.92–8.91%, n = 1 study) had moderate disease^35^ and 40.43% (95% CI 0.05–31,869.21%; n = 14 studies) had severe COVID-19^11,35,46,49,65,70,74,77,79,82,87,89,95,101^ in the IBD population. A significant level of heterogeneity (I^2^: 100%) was observed among the studies, hence a random effect model was used for the analysis (Fig. 6).

The visual inspection of funnel plot observed an obvious asymmetry (Supplementary File S3E) suggestive of publication bias which was confirmed by Egger’s (p = 0.025) and Begg’s (p = 0.0002). The sensitivity analysis was not performed for this analysis.

#### Risk of severe COVID-19 in patients with IBD

A summary estimate of 9 studies^30,31,63,77,79,83,94,99,101^ indicated no association between IBD and severe COVID-19 (OR 1.05; 95% CI 0.73–1.49; p = 0.80) compared to non-IBD patients. A substantial level of heterogeneity (I^2^: 66%) was observed, hence random effect model was applied.

**Figure 7:**
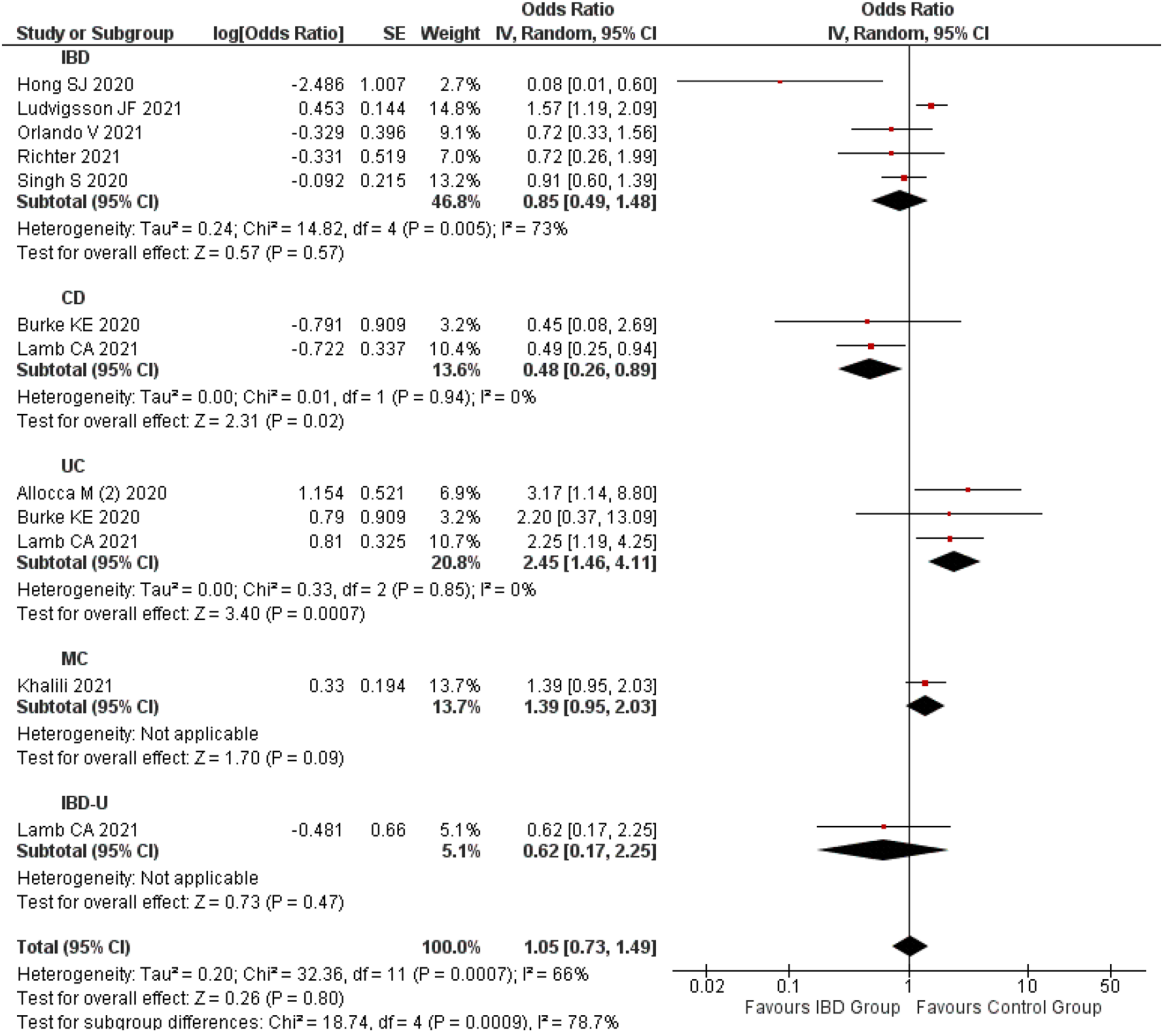
Risk of severe COVID-19 among the patients with IBD.

The subgroup analysis indicated that CD patients^30,79^ had a significantly lesser risk of severe COVID-19 (OR 0.48; 95% CI 0.26–0.89; p = 0.02; 2 studies) while UC patients^30,79,94^ had a significantly higher risk of severe COVID-19 (OR 2.45; 95% CI 1.46–4.11; p < 0.0007; 3 studies). There was non-significant risk of severe COVID-19 with MC (OR 1.39; 95% CI 0.95–2.03; p = 0.09; 1 study)^63^ and IBD-unclassified (IBD-U) (OR 0.62; 95% CI 0.17–2.25; p = 0.47; 1 study)^30^. The heterogeneity observed in the overall analysis was not observed in subgroup analysis based on the type of IBD such as CD and UC (I^2^: 0%). This indicates that type of IBD might be a significant factor that contributed to the variation observed among the study findings (Fig. 7).

The visual inspection of funnel plot observed no obvious asymmetry (Supplementary File S3F) which is suggestive of no publication bias which was confirmed by Egger’s (p = 0.159) and Begg’s (p = 0.272). The sensitivity analysis by removing Burke et al.^79^ indicated no changes in overall results (OR 1.02; 95% CI 0.71–1.47). The result is provided in Supplementary File S4E.

### Mortality in COVID-19 IBD patients

A pooled analysis of 24 studies^25–27,33,34,38,47,48,57,60,65,67,72,74,76,77,79,82,86,89,94,95,101^ estimated an overall mortality rate of 1.94% (95% CI 1.29–2.59%) in COVID-19 patients with IBD. A significant level of heterogeneity (I^2^: 98%) was observed among the studies.

**Figure 8:**
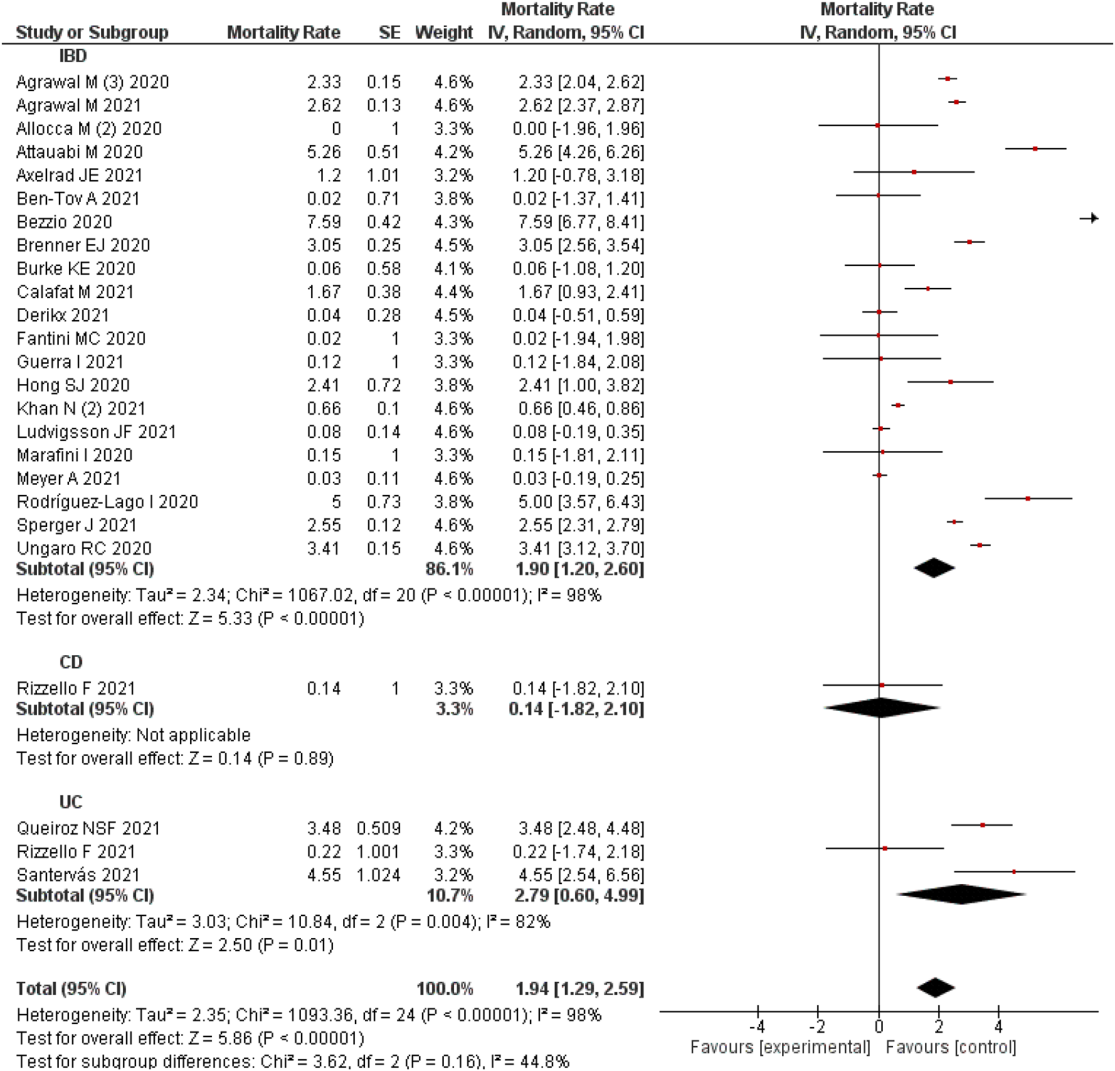
Mortality rate in COVID-19 IBD patients.

Subgroup analysis indicates that, a single study^76^ reported mortality rate of 0.14% (95% CI − 1.82 to 2.10%) in CD patients and an estimate of 3 studies^26,57,76^ indicated a mortality rate of 2.79% (95% CI 0.60–4.99%) in patients with UC. The subgroup analysis based on the type of IBD did not alter the level of heterogeneity indicative of its non-contribution in heterogeneity (Fig. 8).

The visual inspection of funnel plot observed no obvious asymmetry (Supplementary File S3F) which is suggestive of no publication bias which was confirmed by Egger’s (p = 0.348) and Begg’s (p = 0.881). A sensitivity analysis by removing the study by Axelrad et al.^67^ indicated no changes in overall results (OR 1.90; 95% CI 1.30–2.62). The result is provided in Supplementary File S4F.

#### Risk of death or mortality

A meta-analysis of 11 studies^11–13,27,48,50,51,57,62,77,101^ observed that IBD was not significantly associated with COVID-19-related mortality (OR 2.31; 95% CI 0.78–6.81; p = 0.13) compared to non-IBD patients. A random-effect model was used as there was a significant level of heterogeneity (p < 0.10; I^2^: 98%).

**Figure 9:**
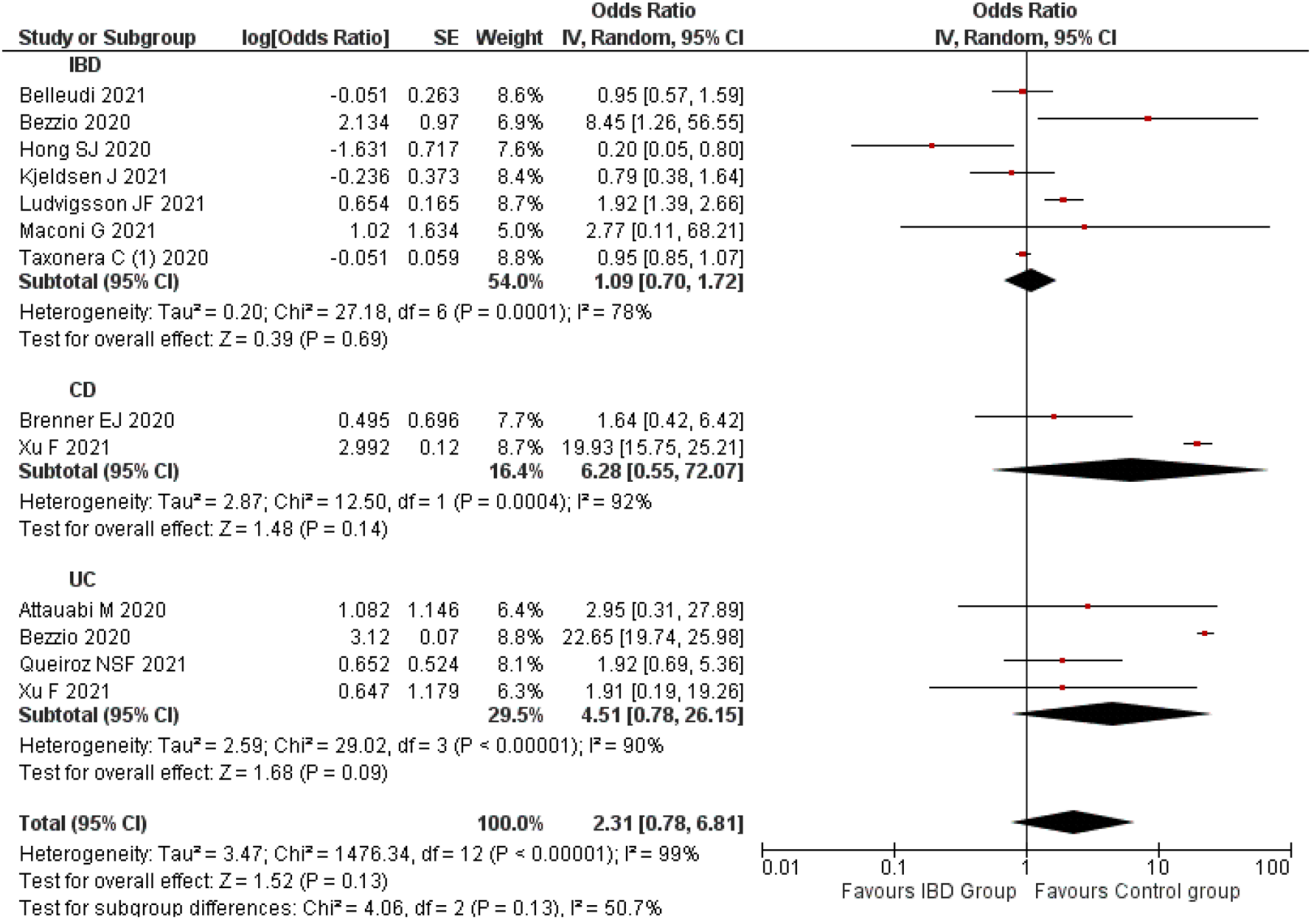
Risk of COVID-19 mortality among the IBD population.

The subgroup analysis also observed the similar non-significant association with CD (OR 6.28; 95% CI 0.55, 72.07; p = 0.14; 2 studies) and UC (OR 4.51; 95% CI 0.78–26.15; p = 0.09; 4 studies) compared to non-CD^48,50^ and non-UC participants^11,27,50,57^, respectively. There was no change in the level of heterogeneity (Fig. 9).

**Figure 10:**
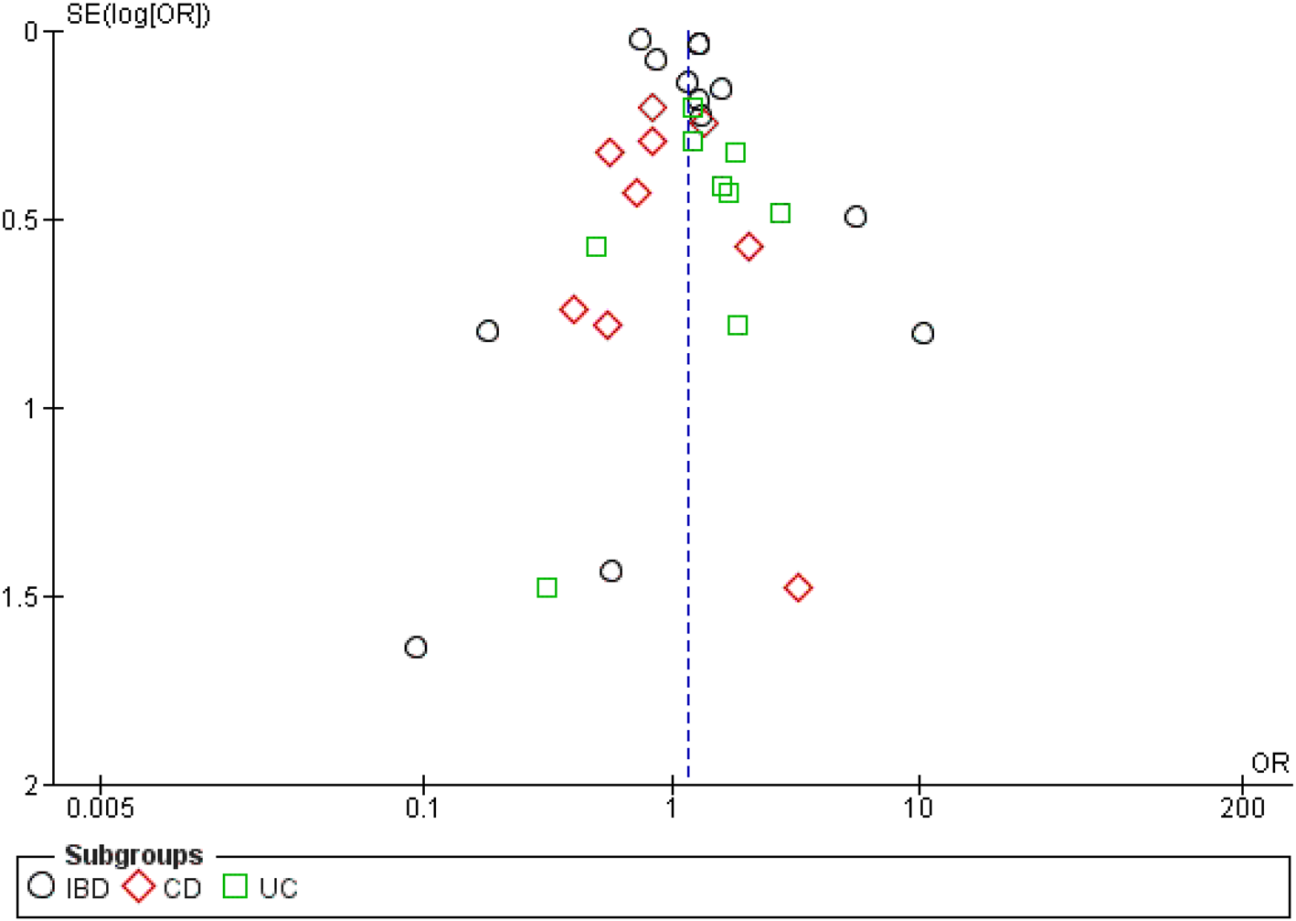
Funnel plot for the risk of COVID-19 outcomes in IBD patients.

The visual inspection of funnel plot observed no obvious asymmetry (Supplementary File S3H) which is suggestive of no publication bias which was confirmed by Egger’s (p = 0.849) and Begg’s (p = 0.881) (Fig. 10). A sensitivity analysis by removing the study by Maconi et al.^13^ indicated no changes in overall results (OR 2.29; 95% CI 0.75–6.94). The result is provided in Supplementary File S4G.

## Discussion

As there is conflicting evidence with respect to the incidence of COVID-19 in patients with IBD, the currently available guidelines for IBD management support the continuation of the use of biologics such as tofacitinib, ustekinumab, and vedolizumab^108^. Moreover, existing evidence fails to establish a positive association between the use of biologics or immunosuppressives with the risk of COVID-19^79^. Additionally, biologics use was associated with a lower risk of COVID-19 hospitalization, intensive care unit admission, and mortality among IBD patients^107^. Most of the studies were from the USA, Italy, and Spain, and the remaining countries were observed to have a lesser number of studies from an IBD population. This is an indication of underreporting, which might be due to a lack of manpower or test kits, and other barriers to access the data and patients^107^.

The findings from the current meta-analysis indicate an overall prevalence of 6.10% (95% CI 3.15–9.04%) of COVID-19 in patients with IBD. Moreover, a significant association could not be identified from the risk estimate (OR 1.15; 95% CI 0.97–1.37; p = 0.11). Similarly, a previous meta-analysis by Singh et al., suggested no difference in risk of COVID-19 in IBD patients when compared to the general population^107^. However, their findings with regards to the association between the risk of COVID-19 and type of IBD differed from our observation. They recorded a non-significant risk in both CD and UC patients, whereas our analysis showed a significantly higher risk in UC patients (OR 1.37; 95% CI 1.07–1.74; p = 0.01). This might be due to the single comparison group that was used by Singh et al., which is the general population. Interestingly, the meta-analysis of 14 studies performed by Tripathi et al., recorded a similar observation in which they recorded a very low incidence (1.01%) of COVID-infection in IBD cohort. Their therapy based analysis revealed a significantly poorer outcomes among those on corticosteroids or mesalamine, though anti-TNFs group had a better outcomes^108^. These findings were strengthened by another meta-analysis conducted by Alrashed et al.^109,110^. They also posed a higher risk with other management such as 5-aminosalicylic acid. However, use of vedolizumab, tofacitinib, and immunomodulators alone or in combination with anti-TNF were not associated with severe disease, rather anti-TNFs, and ustekinumab had a better outcomes. The reported COVID-19 hospitalization rate was 10.63% (95% CI 6.67–14.60%) in patients with IBD, which was lesser in the CD (9.43%; 95% CI − 7.90 to 26.75%) and higher in UC (11.85%; 95% CI − 9.25, 32.95%) subtypes, respectively. A similar trend was observed with the risk of COVID-19 hospitalization, where a non-significant association was found in the overall IBD population (p = 0.50), CD (p = 0.32), and MC (p = 0.11) patients. Moreover, the risk of COVID-19 hospitalization was significantly higher among patients with UC (p < 0.00001). The severe nature of disease, high level of immunosuppression, and higher hospitalization rate might have contributed to a significantly higher rate of COVID-associated hospitalization and severity in patients with UC than CD^107,111^.

We could observe through our meta-analysis that 7.95% and 2.86% of IBD patients had mild and moderate disease, though a higher percentage (40.43%; 95% CI 0.05–31,869.21%) had severe COVID-19. A non-significant association was observed between severe COVID-19 and IBD (p = 0.80), MC (p = 0.09) and IBD-U (p = 0.47). In contrast, a significantly lesser risk was observed in CD patients (OR 0.48; p = 0.02) and significantly higher risk in UC patients (OR 2.45; p < 0.0007). Along with the nature of the disease, factors such as advanced age of ≥ 65 years^72,79^, unvaccinated status^89^, CC subtype, use of oral steroids and proton pump inhibitors, rs13071258 A variant^63^, female gender, obesity, and concomitant diseases such as diabetes, hypertension, and asthma^79^ were associated with a higher risk of severe COVID-19 in IBD patients.

The pooled mortality rate was found to be 1.94%, 0.14%, and 2.79% in IBD, CD, and UC patients respectively. A non-significant association was observed between the COVID-19 mortality and IBD (p = 0.13) and subtypes such as CD (p = 0.14) and UC (p = 0.09). Comparatively, studies by Bezzio et al. (OR 8.45)^11^, and Ludvigsson et al. (OR 1.92)^77^ recorded significantly higher mortality in IBD patients. Similarly, Xu et al.^50^ and Bezzio et al.^11^ recorded significantly higher mortality in CD (OR 19.93) and UC (OR 22.65) patients, respectively. The evidence indicates that many other factors, such as the use of biologics^12^, advanced age^11,42,95^, active IBD status, higher Charlson comorbidity index score^11^, comorbidities, use of corticosteroids^48,101,112^ and thiopurines^101^ were significantly associated with COVID-19 mortality in the IBD population. Very recent evidence also indicates a non-significant effect of corticosteroids in mortality among patients with acute respiratory distress syndrome (ARDS), although positive evidence is reported in more recent randomized clinical trials^113^. Hence, the use of corticosteroids needs to be monitored in the general population as well as in IBD patients with COVID-19 or ARDS.

There was a significant level of heterogeneity observed in the pooled analysis of all outcomes such as the risk of COVID-19, COVID-19 hospitalization, and severe COVID-19, except for the mortality analysis. Through the subgroup analysis, we found that the type of IBD might have contributed to the heterogeneity as the heterogeneity decreased or became non-significant following the subgroup analysis based on the type of IBD. The risk of bias was observed to be lesser in our included studies which indicates a good quality of the studies. Our sensitivity analysis, which was done by removing the studies with the lowest weight, revealed the robustness of our findings by yielding a non-differing result from the original results.

## Conclusions

The current evidence indicates that UC is significantly associated with a higher risk of COVID-19, COVID-19 hospitalization, and severe COVID-19 compared to non-UC participants. Additionally, CD patients had a significantly lesser risk of severe COVID-19 compared to non-CD patients. However, no significant association was observed between higher risk of COVID-19, COVID-19 hospitalization, severe COVID-19, and COVID-19 mortality among those who had been diagnosed non-specifically with IBD compared to non-IBD patients.

## Supplementary Information

Supplementary Information 1.

Supplementary Information 2.

Supplementary Information 3.

Supplementary Information 4.
