# Supplementary material for: Inflammatory bowel disease and COVID-19 outcomes: a meta-analysis
Source: Sci Rep. 2022 Dec 9;12:21333. doi: 10.1038/s41598-022-25429-2 (PMC9734125; doi:10.1038/s41598-022-25429-2)

**Supplementary files S1: Detailed search strategy in various databases**

1a. PubMed Search Strategy

| **Search** | **Query** | **No. of hits** |
| --- | --- | --- |
| #1 | ("Colitis") OR ("Colitis Gravis") OR ("Inflammatory Bowel Disease, Ulcerative Colitis Type") OR ("Crohn's Enteritis") OR ("Regional Enteritis") OR ("Crohn's Disease") OR ("Crohns Disease") OR ("Inflammatory Bowel Disease") OR ("Enteritis, Granulomatous") OR ("Granulomatous Enteritis") OR ("Enteritis") OR ("Regional Ileocolitis") OR ("Colitis, Granulomatous") OR ("Granulomatous Colitis") OR ("Ileitis, Terminal") OR ("Terminal Ileitis") OR ("Ileitis, Regional") OR ("Regional Ileitides") OR ("Regional Ileitis") | 150,992 |
| #2 | “Inflammatory bowel disease” [All Fields] | 112,070 |
| #3 | #1 OR #2 | 159,661 |
| #4 | “COVID 19” OR “ COVID-19 Virus Disease” OR “ COVID 19 Virus Disease” OR “ COVID-19 Virus Diseases” OR “ Disease, COVID-19 Virus” OR “ Virus Disease, COVID-19” OR “ COVID-19 Virus Infection” OR “ COVID 19 Virus Infection” OR “ COVID-19 Virus InfectionsOR Infection, COVID-19 Virus” OR “ Virus Infection, COVID-19” OR “ 2019-nCoV Infection” OR “ 2019 nCoV Infection” OR “ 2019-nCoV Infections” OR “ Infection, 2019-nCoV” OR “ Coronavirus Disease-19” OR “ Coronavirus Disease 19” OR “ 2019 Novel Coronavirus Disease” OR “ 2019 Novel Coronavirus Infection” OR “ 2019-nCoV Disease” OR “ 2019 nCoV Disease” OR “ 2019-nCoV Diseases” OR “ Disease, 2019-nCoV” OR “ COVID19 Coronavirus Disease” OR “ 2019 Disease 2019, Coronavirus” OR “ SARS Coronavirus 2 Infection” OR “ SARS-CoV-2 Infection” OR “ Infection, SARS-CoV-2” OR “ SARS CoV 2 Infection” OR “ SARS-CoV-2 Infections” | 155,681 |
| #5 | #3 AND #4 | 756 |

1b. Scopus Search Strategy

| **Search** | **Query** | **No. of hits** |
| --- | --- | --- |
| #1 | ("Colitis") OR ("Colitis Gravis") OR ("Inflammatory Bowel Disease, Ulcerative Colitis Type") OR ("Crohn's Enteritis") OR ("Regional Enteritis") OR ("Crohn's Disease") OR ("Crohns Disease") OR ("Inflammatory Bowel Disease") OR ("Enteritis, Granulomatous") OR ("Granulomatous Enteritis") OR ("Enteritis") OR ("Regional Ileocolitis") OR ("Colitis, Granulomatous") OR ("Granulomatous Colitis") OR ("Ileitis, Terminal") OR ("Terminal Ileitis") OR ("Ileitis, Regional") OR ("Regional Ileitides") OR ("Regional Ileitis") | 564,837 |
| #2 | “Inflammatory bowel disease” [All Fields] | 253,633 |
| #3 | #1 OR #2 | 564,837 |
| #4 | “COVID 19” OR “ COVID-19 Virus Disease” OR “ COVID 19 Virus Disease” OR “ COVID-19 Virus Diseases” OR “ Disease, COVID-19 Virus” OR “ Virus Disease, COVID-19” OR “ COVID-19 Virus Infection” OR “ COVID 19 Virus Infection” OR “ COVID-19 Virus InfectionsOR Infection, COVID-19 Virus” OR “ Virus Infection, COVID-19” OR “ 2019-nCoV Infection” OR “ 2019 nCoV Infection” OR “ 2019-nCoV Infections” OR “ Infection, 2019-nCoV” OR “ Coronavirus Disease-19” OR “ Coronavirus Disease 19” OR “ 2019 Novel Coronavirus Disease” OR “ 2019 Novel Coronavirus Infection” OR “ 2019-nCoV Disease” OR “ 2019 nCoV Disease” OR “ 2019-nCoV Diseases” OR “ Disease, 2019-nCoV” OR “ COVID19 Coronavirus Disease” OR “ 2019 Disease 2019, Coronavirus” OR “ SARS Coronavirus 2 Infection” OR “ SARS-CoV-2 Infection” OR “ Infection, SARS-CoV-2” OR “ SARS CoV 2 Infection” OR “ SARS-CoV-2 Infections” | 220,515 |
| #5 | #3 AND #4 | 4,039 |
| #6 | #5 AND ( LIMIT-TO ( DOCTYPE , "ar" ) OR LIMIT-TO ( DOCTYPE , "ed" ) OR LIMIT-TO ( DOCTYPE , "sh" ) ) | 1,969 |

1c. Cochrane Search Strategy


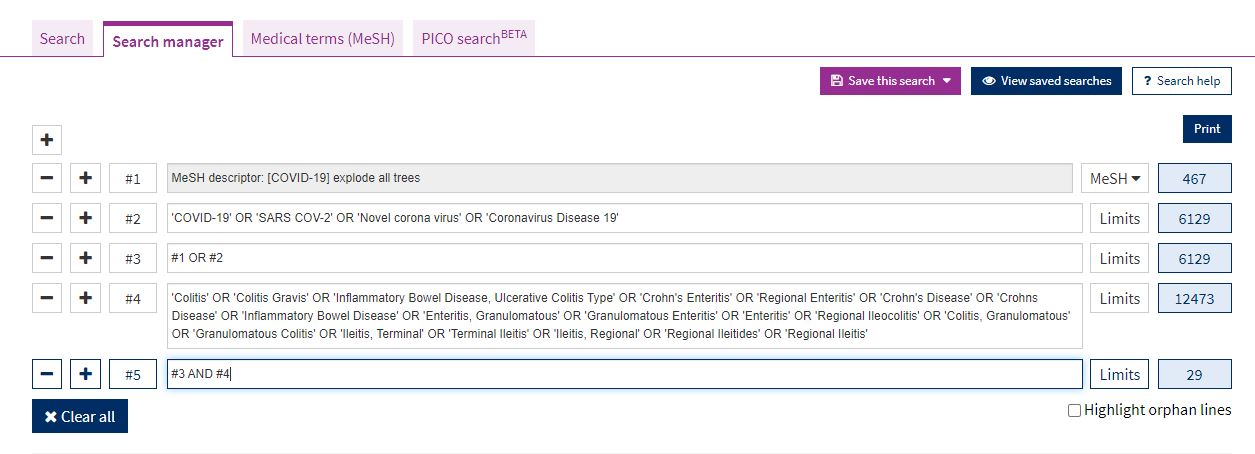


1d. Embase search strategy
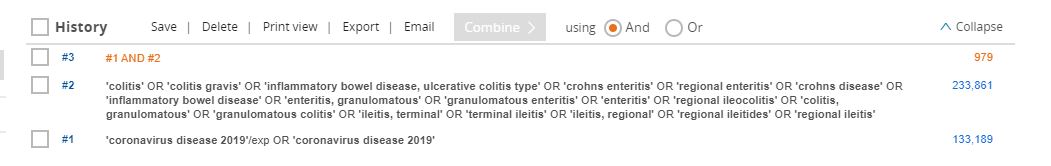

Supplement: Supplementary file 1 — Supplementary Information 1. [file 41598_2022_25429_MOESM1_ESM.docx]
