# Supplementary material for: Inflammatory bowel disease and COVID-19 outcomes: a meta-analysis
Source: Sci Rep. 2022 Dec 9;12:21333. doi: 10.1038/s41598-022-25429-2 (PMC9734125; doi:10.1038/s41598-022-25429-2)
Supplement: Supplementary file 2 — Supplementary Information 2. [file 41598_2022_25429_MOESM2_ESM.docx]

**Supplementary file S2: Risk of bias assessment of included studies**

***S2A. Risk of bias in prevalence studies***

| **Study ID** | **Was the sample frame appropriate to address the target population?** | **Were study participants sampled in an appropriate way?** | **Was the sample size adequate?** | **Were the study subjects and the setting described in detail?** | **Was the data analysis conducted with sufficient coverage of the identified sample?** | **Were valid methods used for the identification of the condition?** | **Was the condition measured in a standard, reliable way for all participants?** | **Was there appropriate statistical analysis?** | **Was the response rate adequate, and if not, was the low response rate managed appropriately?** |
| --- | --- | --- | --- | --- | --- | --- | --- | --- | --- |
| Marafini I et al., | Yes | Yes | Yes | Yes | Yes | Yes | Yes | Yes | NA |
| Santervás et al., | Yes | Yes | Unclear | Yes | No | Unclear | Unclear | No | Yes |
| Ferrer et al., | Yes | Yes | Yes | Yes | Unclear | Unclear | Unclear | No | Yes |
| Markovic S et al., | Yes | Yes | Yes | Yes | No | Yes | Yes | Yes | Yes |
| Botwin G et al., | Yes | Yes | Yes | Yes | Yes | Yes | Yes | Yes | Yes |
| Refaie et al., | Yes | Yes | Yes | Yes | Yes | Yes | Yes | Yes | Yes |
| Navarro-Correal et al., | Yes | Yes | Yes | Yes | Yes | Yes | Yes | Yes | Yes |
| Allocca M (1) et al., | Yes | Yes | Yes | Yes | Yes | Yes | Yes | Yes | Yes |
| Scaldaferri F et al., | Yes | Yes | Yes | Yes | Yes | Yes | Yes | Yes | Yes |
| Quera R et al., | Yes | Yes | Yes | Yes | Yes | Yes | Yes | Yes | Yes |
| Vadan R et al., | Yes | Yes | Yes | Yes | Yes | Yes | Yes | Yes | Yes |
| Anushiravani A et al., | Yes | Yes | Yes | Yes | Yes | Yes | Yes | Yes | Yes |
| Taxonera C (2) et al., | Yes | Yes | Yes | Yes | Yes | Yes | Yes | Yes | Yes |
| Axelrad JE et al., | Yes | Yes | Yes | Yes | Yes | Unclear | Yes | Yes | Yes |
| Dailey J et al., | Yes | Yes | Yes | Yes | Yes | Yes | Yes | Yes | Yes |
| Iborra I et al., | Yes | Yes | Yes | Yes | Yes | Yes | Yes | Yes | Yes |
| El Hajra et al., | Yes | Yes | Yes | Yes | Yes | Yes | Yes | Yes | Yes |
| Scucchi L et al., | Yes | Yes | Yes | Yes | Yes | Yes | Yes | Yes | Yes |
| Carparelli et al., | Yes | Yes | Yes | Yes | Yes | Yes | Yes | Yes | Yes |
| Harris RJ et al., | Yes | Yes | Yes | Yes | Yes | Unclear | Unclear | Yes | Yes |
| Bezzio C et al., | Yes | Yes | Yes | Yes | Yes | Yes | Yes | Yes | Yes |
| Lewine E et al., | Yes | Yes | Yes | Yes | Yes | Yes | Yes | Yes | Yes |
| Hormati A et al. | Yes | Yes | Yes | Yes | Yes | Yes | Yes | Yes | Yes |
| An P et al., | Yes | Yes | Yes | Yes | Yes | Yes | Yes | Yes | Yes |

***S2B. Risk of bias in Cross sectional studies***

| **Study ID** | **Were the criteria for inclusion in the sample clearly defined?** | **Were the study subjects and the setting described in detail?** | **Was the exposure measured in a valid and reliable way?** | **Were objective, standard criteria used for measurement of the condition?** | **Were confounding factors identified?** | **Were strategies to deal with confounding factors stated?** | **Were the outcomes measured in a valid and reliable way?** | **Was appropriate statistical analysis used?** |
| --- | --- | --- | --- | --- | --- | --- | --- | --- |
| Moum et al., | Yes | Yes | Yes | Yes | Yes | Unclear | Yes | Yes |
| Richter et al., | Yes | Yes | Yes | Yes | Yes | Yes | Yes | Yes |
| Caron B et al., | Yes | Yes | Yes | Yes | No | No | Yes | Yes |
| Sperger J et al., | Yes | Yes | Yes | Yes | Yes | Yes | Yes | Yes |
| Mahmud N et al., | Yes | Yes | Yes | Yes | Yes | Yes | Yes | Yes |
| Álvarez PF et al., | Yes | Yes | Yes | Yes | Yes | Yes | Yes | Yes |
| Rodríguez-Lago I et al., | Yes | Yes | Yes | Yes | No | No | Yes | Yes |
| Brenner EJ et al., | Yes | Yes | Yes | Yes | Yes | Yes | Yes | Yes |
| Taxonera C (1) et al., | Yes | Yes | Yes | Yes | Yes | Yes | Yes | Yes |
| Femury M et al., | Yes | Yes | Yes | Yes | No | No | Yes | Yes |
| Xu F et al., | Yes | Yes | Yes | Yes | Yes | Yes | Yes | Yes |
| Queiroz NSF et al., | Yes | Yes | Yes | Yes | Yes | Yes | Yes | Yes |
| Crispino et al., | Yes | Yes | Yes | Yes | Yes | Yes | Yes | Yes |
| Agrawal M (1) et al., | Yes | Yes | Yes | Yes | Yes | Yes | Yes | Yes |
| Askar SR et al., | Yes | Yes | Yes | Yes | Yes | Yes | Yes | Yes |
| Agrawal M (1) et al., | Yes | Yes | Yes | Yes | Yes | Yes | Yes | Yes |
| Ghoshal UC et al., | Yes | Yes | Yes | Yes | Yes | Yes | Yes | Yes |
| Agrawal M (2) et al., | Yes | Yes | Yes | Yes | Yes | Yes | Yes | Yes |
| Agrawal M (3) et al., | Yes | Yes | Yes | Yes | Yes | Yes | Yes | Yes |
| Ungaro RC et al., | Yes | Yes | Yes | Yes | Yes | Yes | Yes | Yes |
| Schlabitz F et al., | Yes | Yes | Yes | Yes | No | No | Yes | Yes |
| Guerra I et al., | Yes | Yes | Yes | Yes | Yes | Yes | Yes | Yes |
| Parekh R et al., | Yes | Yes | Yes | Yes | Yes | Yes | Yes | Yes |
| Dalal RS et al., | Yes | Yes | Yes | Yes | Yes | Yes | Yes | Yes |
| Opheim R et al., | Yes | Yes | Yes | Unclear | No | No | No | Yes |
| Fantini MC et al., | Yes | Yes | Yes | Yes | No | No | Yes | Yes |
| Allocca M (3) et al., | Yes | Yes | Yes | Yes | No | No | Yes | Yes |
| Mosli M et al., | Yes | Yes | Yes | Yes | Yes | Yes | Yes | Yes |
| Grunert PC et al., | Yes | Yes | Yes | Yes | No | No | Yes | Yes |

***S2C. Risk of bias in Cohort studies***

| **Study** | **Were the two groups similar and recruited from the same population?** | **Were the exposures measured similarly to assign people to both exposed and unexposed groups?** | **Was the exposure measured in a valid and reliable way?** | **Were confounding factors identified?** | **Were strategies to deal with confounding factors stated?** | **Were the groups/participants free of the outcome at the start of the study (or at the moment of exposure)?** | **Were the outcomes measured in a valid and reliable way?** | **Was the follow up time reported and sufficient to be long enough for outcomes to occur?** | **Was follow up complete, and if not, were the reasons to loss to follow up described and explored?** | **Were strategies to address incomplete follow up utilized?** | **Was appropriate statistical analysis used?** |
| --- | --- | --- | --- | --- | --- | --- | --- | --- | --- | --- | --- |
| Attauabi et al., | Yes | Yes | Yes | Yes | Yes | Yes | Yes | Yes | Yes | NA | Yes |
| Lamb CA et al., | Yes | Yes | Yes | Yes | Yes | Yes | Yes | Yes | Yes | NA | Yes |
| Belleudi V et al., | Yes | Yes | Yes | Yes | Yes | Yes | Yes | Yes | Yes | NA | Yes |
| Ben-Tov A et al., | Yes | Yes | Yes | Yes | Yes | Yes | Yes | Yes | Yes | NA | Yes |
| Meyer A et al., | Yes | Yes | Yes | Yes | Yes | Yes | Yes | Yes | Yes | NA | Yes |
| Berte R et al., | Yes | Yes | Yes | No | No | Yes | Yes | Yes | Yes | NA | Yes |
| Khan N (1) et al., | Yes | Yes | Yes | No | No | Yes | Yes | Yes | Yes | NA | Yes |
| Rottoli M et al., | Yes | Yes | Yes | Yes | Yes | Yes | Yes | Yes | Yes | NA | Yes |
| Newsome RC et al., | Yes | Yes | Yes | Yes | Yes | Yes | Yes | Yes | Yes | NA | Yes |
|  |  |  |  |  |  |  |  |  |  |  |  |
| Gubatan J et al., | Yes | Yes | Yes | Yes | Yes | Yes | Yes | Yes | Yes | NA | Yes |
| Bezzio et al., 2020 | Yes | Yes | Yes | Yes | Yes | Yes | Yes | Yes | Yes | NA | Yes |
| Ardizzone S et al., | Yes | Yes | Yes | Yes | Yes | Yes | Yes | Yes | Yes | NA | Yes |
| Hadi YB et al., | Yes | Yes | Yes | Yes | Yes | Yes | Yes | Yes | Yes | NA | Yes |
|  |  |  |  |  |  |  |  |  |  |  |  |
| Kjeldsen J et al., | Yes | Yes | Yes | Yes | Yes | Yes | Yes | Yes | Yes | NA | Yes |
| Kennedy NA et al., | Yes | Yes | Yes | Yes | Yes | Yes | Yes | Yes | Yes | NA | Yes |
| Derikx et al., 2021 | Yes | Yes | Yes | Yes | Yes | Yes | Yes | Yes | Yes | NA | Yes |
| Attauabi M et al., | Yes | Yes | Yes | Yes | Yes | Yes | Yes | Yes | Yes | NA | Yes |
| Łodyga M et al., | Yes | Yes | Yes | Yes | Yes | Yes | Yes | Yes | Yes | NA | Yes |
| Rizzello F et al., | Yes | Yes | Yes | Yes | Yes | Yes | Yes | Yes | Yes | NA | Yes |
| Maconi G et al., | Yes | Yes | Yes | Yes | Yes | Yes | Yes | Yes | Yes | NA | Yes |
| Ludvigsson JF et al., | Yes | Yes | Yes | Yes | Yes | Yes | Yes | Yes | Yes | NA | Yes |
| Burke KE et al. | Yes | Yes | Yes | Yes | Yes | Yes | Yes | Yes | Yes | NA | Yes |
| Calafat M et al., | Yes | Yes | Yes | No | No | Yes | Yes | Yes | Yes | NA | Yes |
| Khan N (2) et al., | Yes | Yes | Yes | Yes | Yes | Yes | Yes | Yes | Yes | NA | Yes |
| Khan N (3) et al., | Yes | Yes | Yes | Yes | Yes | Yes | Yes | Yes | Yes | NA | Yes |
| Norsa L et al., | Yes | Yes | Yes | Yes | Yes | Yes | Yes | Yes | Yes | NA | Yes |
| Allocca M (2) et al., | Yes | Yes | Yes | Yes | Yes | Yes | Yes | Yes | Yes | NA | Yes |
| Viganò C et al., | Yes | Yes | Yes | Yes | Yes | Yes | Yes | Yes | Yes | NA | Yes |
| Lukin DJ et al., | Yes | Yes | Yes | Yes | Yes | Yes | Yes | Yes | Yes | NA | Yes |
| Singh S et al., | Yes | Yes | Yes | Yes | Yes | Yes | Yes | Yes | Yes | NA | Yes |
| Hong SJ et al., | Yes | Yes | Yes | Yes | Yes | Yes | Yes | Yes | Yes | NA | Yes |

***S2C. Risk of bias in Case-control studies***

| **Study** | **Were the groups comparable other than the presence of disease in cases or the absence of disease in controls?** | **Were cases and controls matched appropriately?** | **Were the same criteria used for identification of cases and controls?** | **Was exposure measured in a standard, valid and reliable way?** | **Was exposure measured in the same way for cases and controls?** | **Were confounding factors identified?** | **Were strategies to deal with confounding factors stated?** | **Were outcomes assessed in a standard, valid and reliable way for cases and controls?** | **Was the exposure period of interest long enough to be meaningful?** | **Was appropriate statistical analysis used?** |
| --- | --- | --- | --- | --- | --- | --- | --- | --- | --- | --- |
| Khalili et al., | Yes | Yes | Yes | Yes | Yes | Yes | Yes | Yes | Yes | Yes |
| Orlando V et al., | Yes | Yes | Yes | Yes | Yes | Yes | Yes | Yes | Yes | Yes |
