# Supplementary material for: Inflammatory bowel disease and COVID-19 outcomes: a meta-analysis
Source: Sci Rep. 2022 Dec 9;12:21333. doi: 10.1038/s41598-022-25429-2 (PMC9734125; doi:10.1038/s41598-022-25429-2)
Supplement: Supplementary file 3 — Supplementary Information 3. [file 41598_2022_25429_MOESM3_ESM.docx]

**Supplementary file S3: Funnel plot for the risk of COVID-19 outcomes in IBD patients.**

**
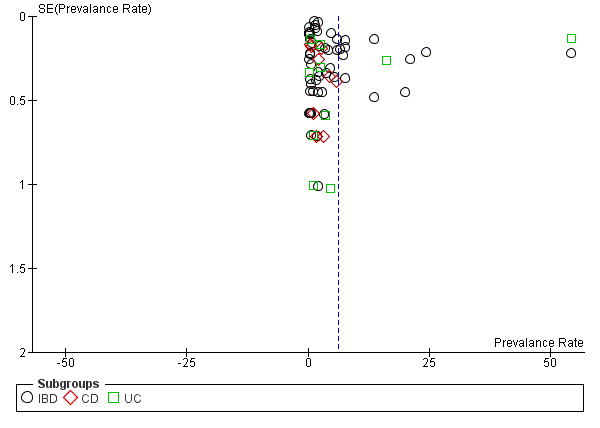
**

***S3A: Prevalence of COVID-19***

**
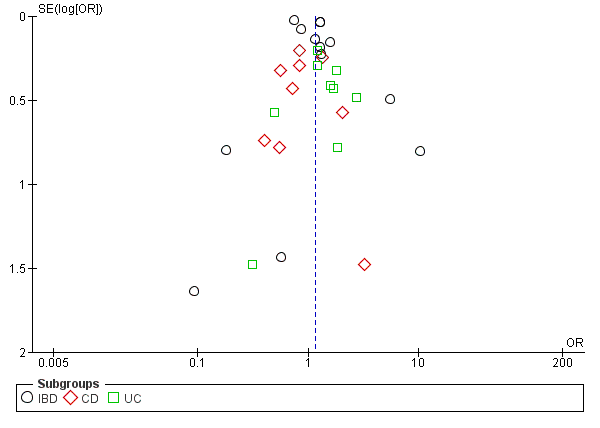
**

***S3B: Risk of COVID-19***

**
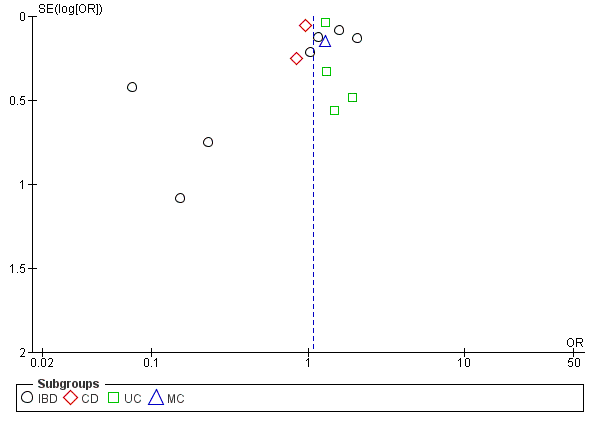
**

***Supplementary file S4C: COVID-19 associated hospitalization prevalence***

***
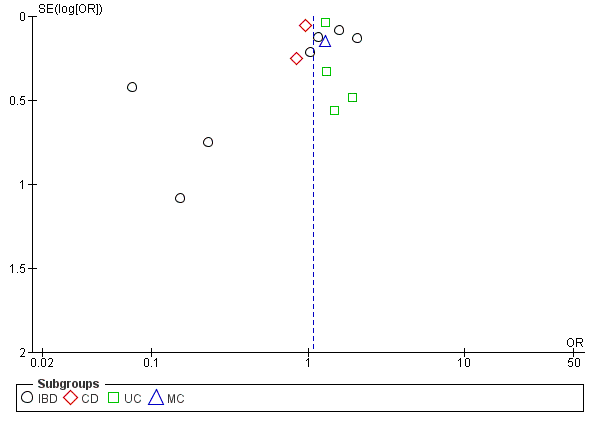
***

***Supplementary file S3D: Risk of COVID-19 associated hospitalization***

***
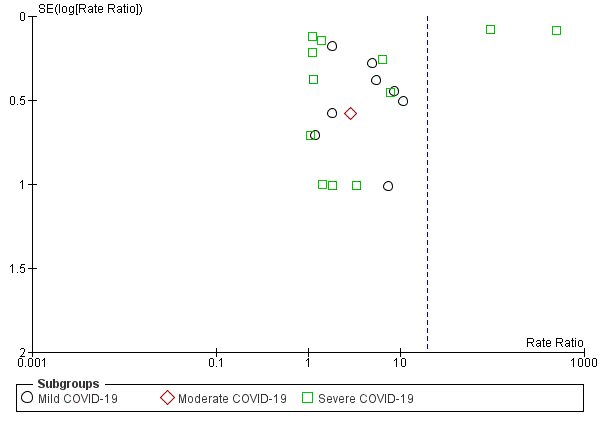
***

***Supplementary file S3E: Severity of COVID-19***

***
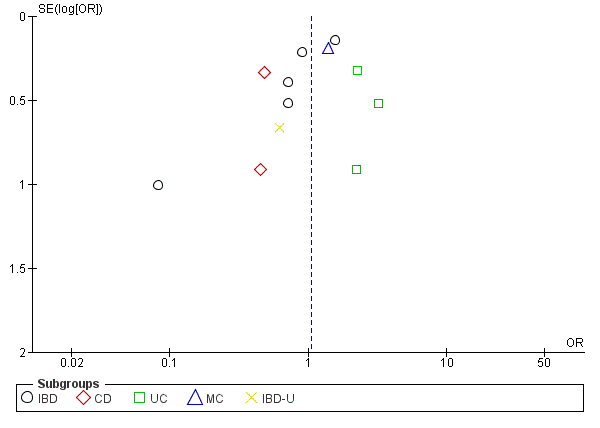
***

***Supplementary file S3F: Risk of Severe COVID-19***

***
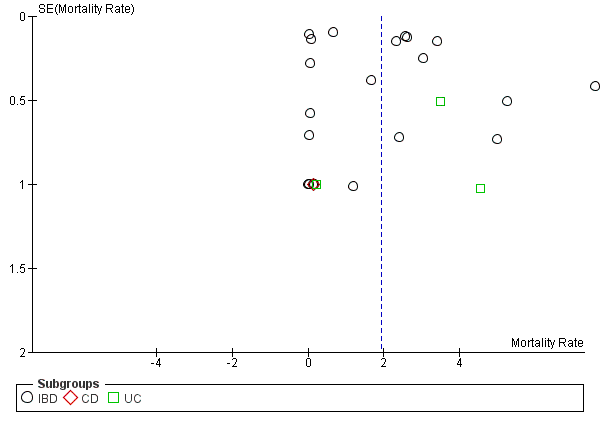
***

***Supplementary file S3G: COVID-19 mortality***


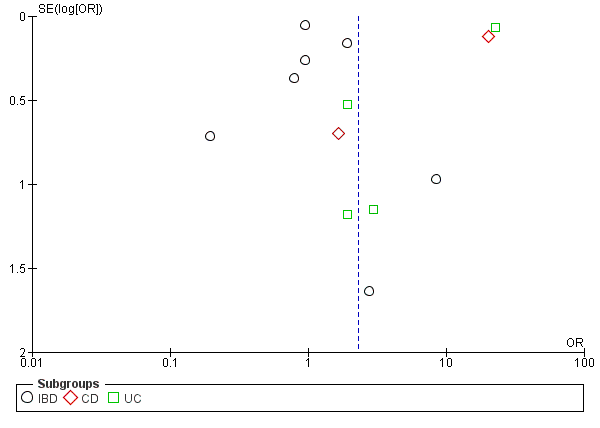


***Supplementary file S3H: Risk of COVID-19 mortality***
