# Supplementary material for: Inflammatory bowel disease and COVID-19 outcomes: a meta-analysis
Source: Sci Rep. 2022 Dec 9;12:21333. doi: 10.1038/s41598-022-25429-2 (PMC9734125; doi:10.1038/s41598-022-25429-2)
Supplement: Supplementary file 4 — Supplementary Information 4. [file 41598_2022_25429_MOESM4_ESM.docx]

**Supplementary file S4: Sensitivity analysis for COVID-19 outcomes in IBD patients**


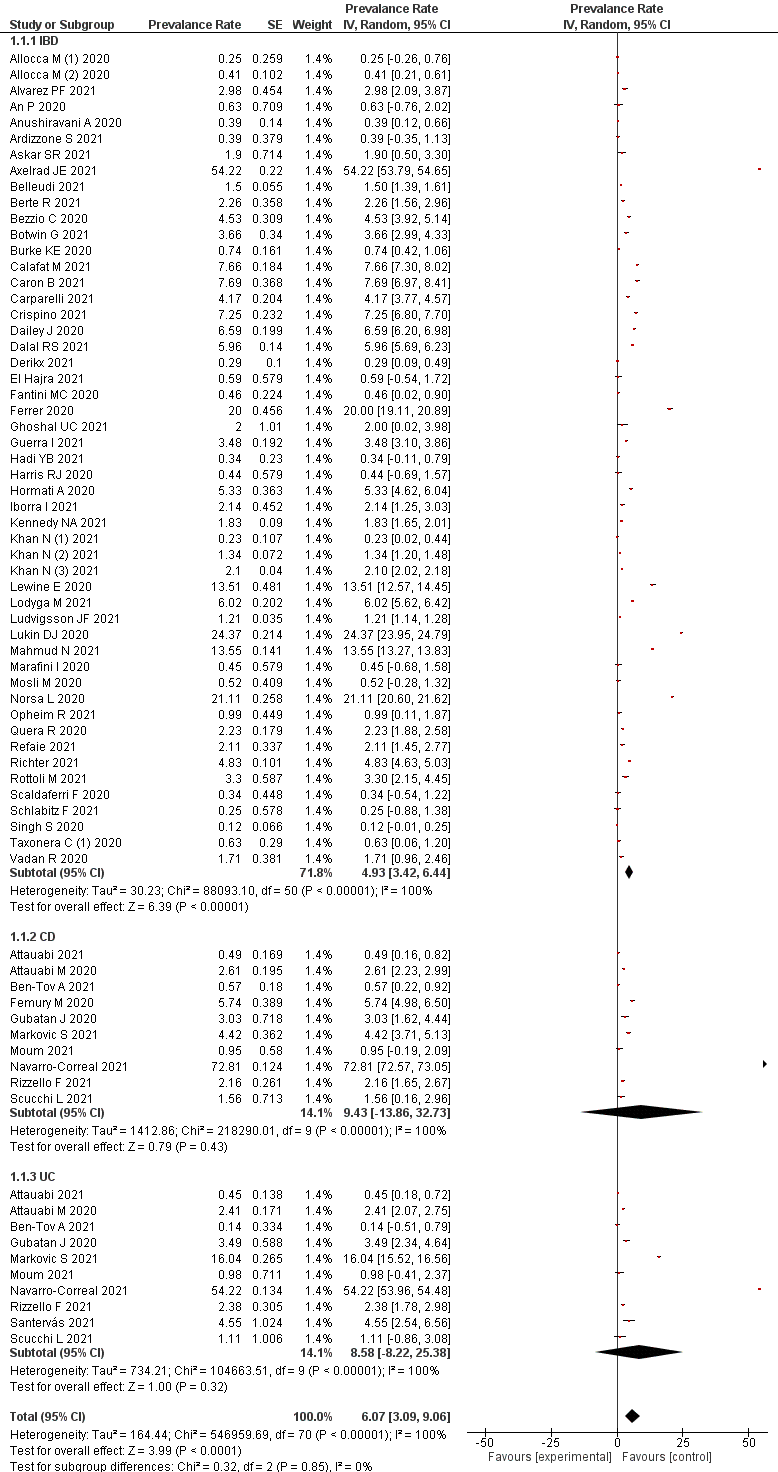


***S4A: Pooled prevalance of COVID-19***


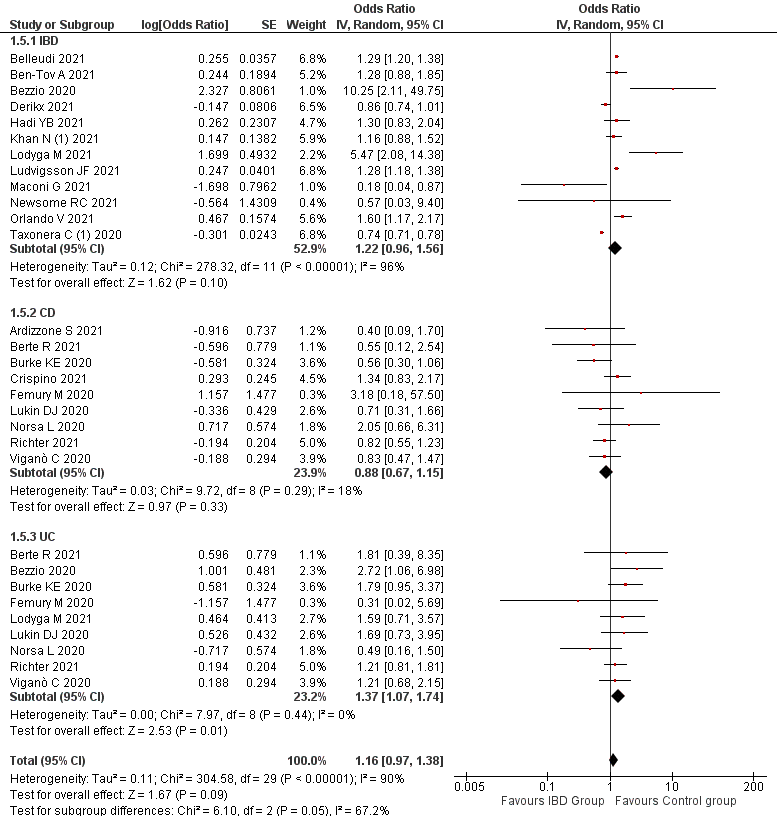


***S4B: Risk of COVID-19***


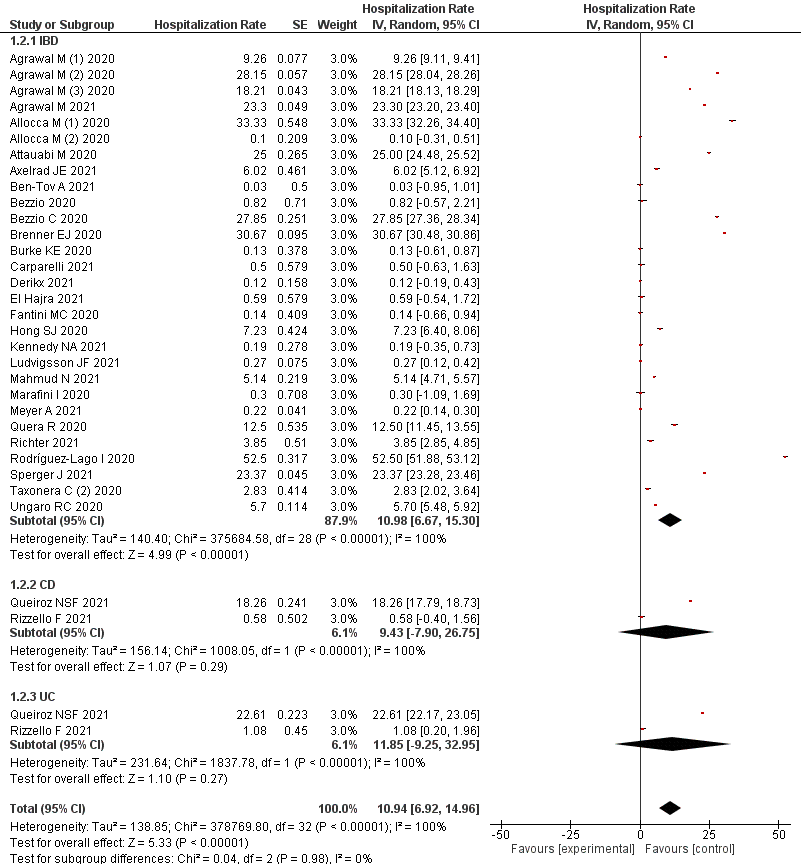


***Supplementary file S4C: COVID-19 associated hospitalization prevalence***

***
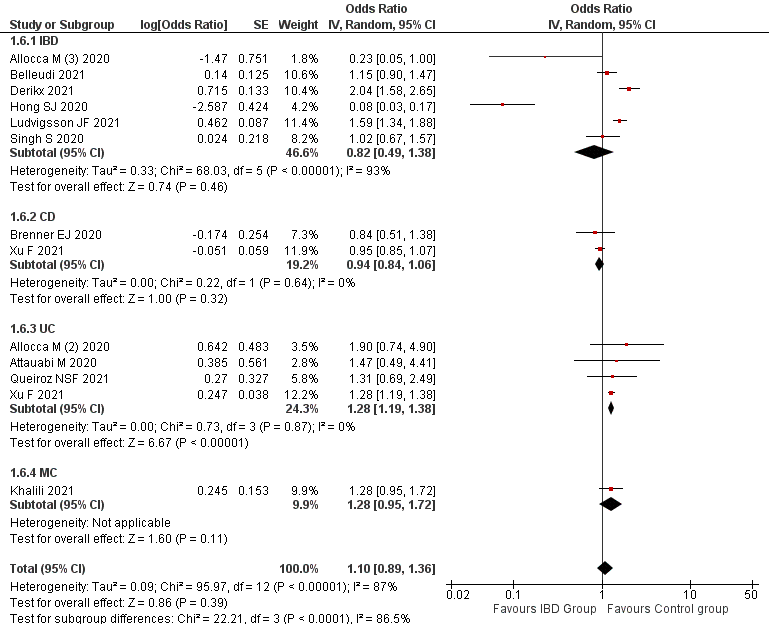
***

***Supplementary file S4D: Risk of COVID-19 associated hospitalization***


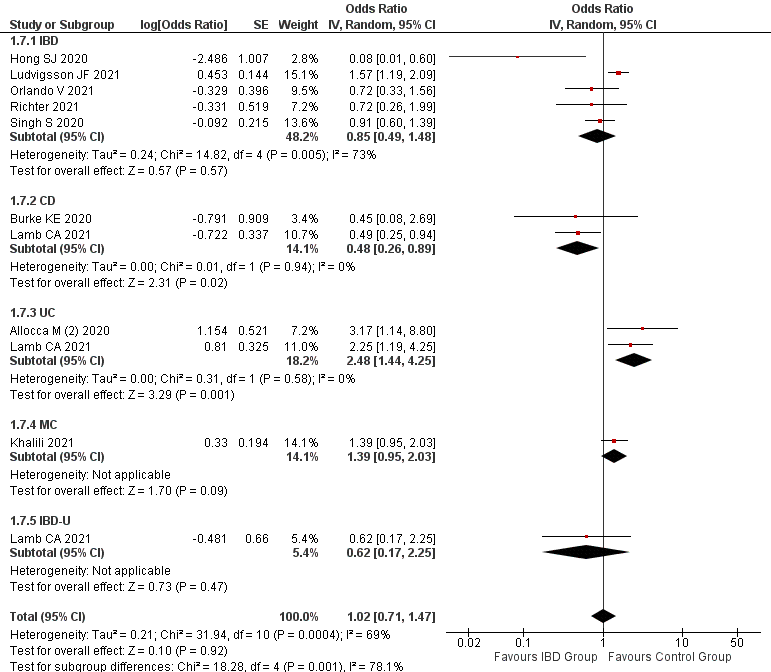


***Supplementary file S4E: Risk of severe COVID-19***

***
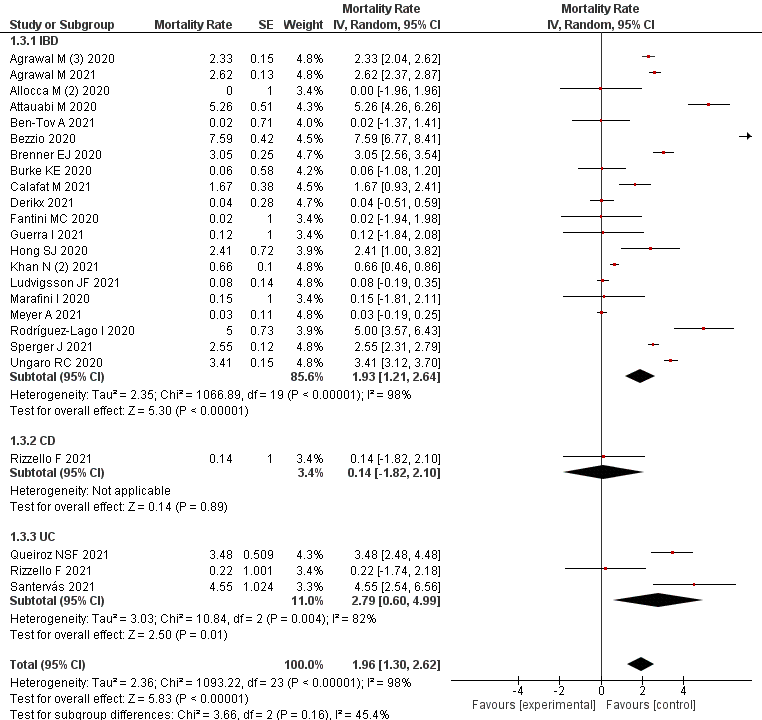
***

***Supplementary file S4F: COVID-19 mortality***


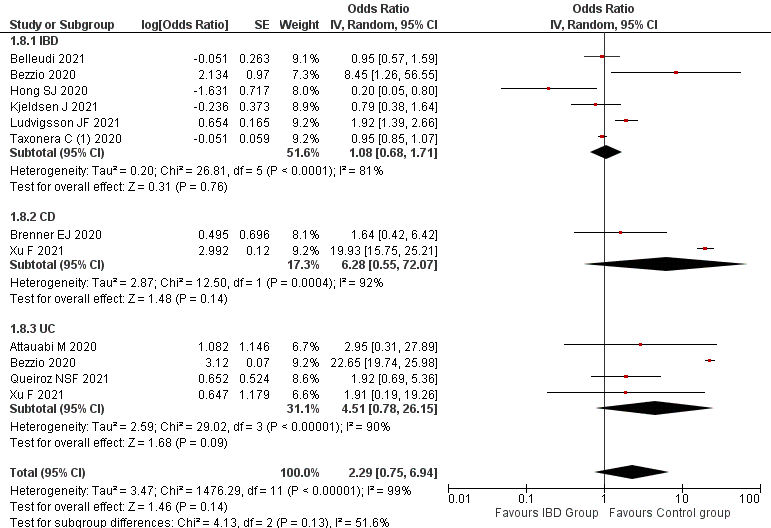


***Supplementary file S4G: Risk of COVID-19 mortality***
